# Effects of Housing Density in Five Inbred Strains of Mice

**DOI:** 10.1371/journal.pone.0090012

**Published:** 2014-03-21

**Authors:** Judith L. Morgan, Karen L. Svenson, Jeffrey P. Lake, Weidong Zhang, Timothy M. Stearns, Michael A. Marion, Luanne L. Peters, Beverly Paigen, Leah Rae Donahue

**Affiliations:** The Jackson Laboratory, Bar Harbor, Maine, United States of America; IGBMC/ICS, France

## Abstract

To evaluate the effect of increased mouse density in a cage, mice were housed at the density recommended by the 1996 *Guide for the Care and Use of Laboratory Animals* and at densities that were approximately 2, 2.6, and 3 times greater. Five strains of mice (129S1/SvImJ, A/J, BALB/cByJ, C57BL/6J, and DBA/2J) were evaluated throughout 3- and 8-month timeframes for health and well-being, including mortality, cardiac measures, plasma cholesterol, body weight, bone mineral density, organ weights, hematology, behavioral observations, and open field and light–dark tests. For 22 of the 27 traits measured, increased housing density had no significant effect. Kidney weight, adrenal weight, and heart rate decreased as mice were housed more densely, and some of the decreases were statistically significant. Reduced kidney weight, adrenal weight, and heart rate are not considered to be negative outcomes and may even indicate reduced stress. However, all measurements of these three traits were within normal physiological ranges. Percent fat increased slightly in strains 129S1/SvImJ, A/J, and DBA/2J, but did not increase in strains BALB/cByJ, and C57BL/6J. These results indicate that mice can be housed at higher densities than those currently recommended.

## Introduction

To ensure the humane treatment of research animals, the *Guide for the Care and Use of Laboratory Animals* (*Guide*) [1] specified the amount of space that should be allocated to each mouse in a cage: an adult mouse of 15–25 gm required at least 77.4 cm^2^ (12 in^2^) and a mouse over 25 gm required 98.6 cm^2^ (15 in^2^). These standards for the density of housing mice were based on the best professional judgment because experimental data were insufficient. However, the *Guide* recognized the paucity of information available to support these guidelines and encouraged alternatives as long as they were data-driven and based on sound science. In the last few decades, a number of improvements have been made in mouse husbandry and caging. Most animal facilities are now considerably cleaner and many are specific pathogen free. Widespread use of individually ventilated cages provides animals with a drier cage and better air quality. The importance of bedding material and thermoregulation of mice is better understood. All these improvements suggested a re-evaluation of housing density recommendations.

After the *Guide* was published in 1996, a number of studies evaluated mice at densities greater than those recommended by the *Guide* [2]–[15]; these studies have been reviewed in several reports [16]–[18]. Reasoning primarily from anthropomorphic considerations, it was generally assumed that animals would desire more space rather than less, and the regulations for animal research in Europe were revised to increase the amount of space available. However, scientific evidence was lacking as to whether more space was either beneficial or preferred by mice. Van Loo and colleagues [14], [15] reported that mice are less aggressive when the floor space per mouse was reduced, particularly for male mice. This reduced aggression led to better survival for mice housed more densely in at least two studies [3], [7]. This reduced aggression was observed in studies where the numbers of bite wounds were recorded; the mice housed more densely had fewer wounds [8]. Several studies also showed that mice were less stressed when housed more densely, as assessed by mortality, behavior, immune function, adrenal weight, and heart rate [2], [3], [7], [10], [14], [19]. Overall, the studies that examined the effects of housing animals at different densities concur that mice can be housed at densities that are about twice that currently suggested by the *Guide*. One confounding problem with the studies is that greater density was generally achieved by putting more mice in a standard cage; this not only changed the density but also changed the size of the social group. However, at least three studies kept the number of mice constant and achieved greater density by changing the size of the cage [3], [7], [14], [15]; these studies reached the same conclusion, namely that mice can be housed at about twice the density recommended by the *Guide*.

Although these studies challenged the space recommendations in the *Guide*, they have not as yet led to changes in the recommendations in the current *Guide* [20], most likely because several of the studies have some limitation. Often the studies used only one or two strains of mice, and several studies used only one sex. However, taken together, a number of strains have now been tested, including four inbred strains — C57BL/6J, BALB/cByJ, FVB/NJ, NOD/ShiLtJ — and the randomly bred ICR and outbred MF1 and Oncins Franc 1. Some studies were underpowered due to the low number of mice tested. Finally, the parameters used to measure well-being varied considerably from study to study.

In this study, we sought to expand the scope of earlier studies. We increased the number of strains to five in common use: 129S1/SvImJ (129), A/J, BALB/cByJ (BALB), C57BL/6J (B6), and DBA/2J (DBA). We aimed to increase the density until we observed an adverse effect, so our highest density was about triple the recommendation, which is more dense than in most previous studies. The lowest density approximated the recommendation from the *Guide*; the other three densities were about 2.0-, 2.6-, and 3.0-fold greater. In addition, our study also evaluated effects on both sexes, used two commonly-used cage types (duplex and shoebox), and included measurements in mice housed for up to 8-months. We tested 27 parameters: mortality, cardiac measures, plasma cholesterol, body composition, organ weights, hematology, and behavioral observations, including open field and light–dark tests, in 9240 mice. The results concur with most previous studies; mice may be housed more densely than currently recommended.

## Materials and Methods

### Mice

Female and male mice of five strains were obtained at wean age from The Jackson Laboratory (JAX): 129S1/SvImJ (129), A/J, BALB/cByJ (BALB), C57BL/6J (B6), and DBA/2J (DBA). Mice were provided *ad libitum* access to autoclaved acidified water (pH 2.8 to 3.1) and fed autoclaved standard laboratory chow containing 6% fat by weight (LabDiet 5K52, St. Louis, MO). The animal room was supplied with HEPA-filtered air at 19 air changes/hour and maintained at 22±2°C, a humidity of 35%±4%, and a 12∶12 hour light∶dark cycle. Bedding, changed weekly, was autoclaved pine shavings (Crobb Box, Ellsworth, ME). The specific pathogen free animal room was monitored for and found free of 15 viruses, 17 bacterial species, two *Mycoplasma* spp., external and internal parasites, and *Encephalitozoon cuniculi* [10].

### Study Protocol

The study included two types of individually ventilated caging from Thoren Caging Systems, Inc. (Hazelton, PA): the “shoebox” cage in common use (Thoren cage #5; 503.7 cm^2^ [78.1 in^2^] floor area) and a duplex cage used at JAX (Thoren cage #3, consisting two separate cages, each with 333.6 cm^2^ [51.6 in^2^] floor area). The dimensions for the #5 cage are (width×length×height) 22.2×30.80×16.24 cm (8.75×12.125×6.395 in) and for the #3 cage are 30.80×30.80×14.05 cm (12.125×12.125×5.53 in). The ventilated caging provided a minimum of 60 air changes per hour within the mouse cage. One group of mice was housed for 3 months and a second group was housed for 8 months. For each group, mice were housed at four different densities in two cage types. The numbers of mice per cage, the housing densities, and the total numbers of mice are provided in Table 1. The greater number of mice in the shoebox cages was necessary to produce a comparable density between the smaller duplex and larger shoebox cages. For each cage type/density/strain/sex group, six cages served as replicates. Mice were randomly assigned to the density groups. All procedures were approved by the Institutional Animal Care and Use Committee and are consistent with the United States Public Health Policy on the Humane Care and Use of Laboratory Animals. Mice were euthanized by CO_2_ exposure.

**Table 1:** Density and floor space according to cage type^a^.

	Duplex cage			Shoebox cage		
Density group	Floor space per mouse		Mice per cage	Floor space per mouse		Mice per cage
	cm^2^	in^2^		cm^2^	in^2^	
**1**	83.2	12.9	4	83.9	13.0	6
**2**	47.7	7.4	7	50.3	7.8	10
**3**	36.8	5.7	9	36.1	5.6	14
**4**	30.3	4.7	11	31.6	4.9	16
Total mice per cage type			3,720			5,520
Total number of cages			480			480

a The duplex cage consists of two separate cages; each has a floor area of 333.6 cm^2^ (51.6 in^2^). The shoebox cage has a floor area of 503.7 cm^2^ (78.1 in^2^).

The housing density of Density group 1 is almost equal to that recommended by the *Guide*, which is 77.4 cm^2^ for mice between 15 and 25 g. Housing densities of groups 2, 3, and 4 are approximately 2.0-, 2.6-, and 3-fold greater. Numbers of mice were chosen to produce nearly equivalent densities between the two cage types. To complete replicate sets of cage densities, mice were obtained in groups of 31 for duplex cages and in groups of 46 for shoebox cages.

Table 2 provides the protocol schedule and age of the mice at each test. Although these methods were described previously [21]–[23], we provide a brief summary here. Blood counts were determined in EDTA-anticoagulated blood using an Advia 120 Multispecies Hematology Analyzer (Bayer Diagnostics, Tarrytown, NY) as described previously [21]. Following a 4-hour food deprivation period from 0700 to 1100 hours, plasma lipids were analyzed in EDTA-anticoagulated blood using a Beckman Coulter Syncron CX5 Delta Chemistry Analyzer according to the manufacturer's instructions (Beckman Coulter, Fullerton, CA). Systolic blood pressure was measured using the Visitech BP2000 system (Visitech Systems, Apex, NC) as described previously [22]. Un-anesthetized mice were restrained with their tails held across sensors that detect blood flow. A computer recorded 20–30 measurements over a 20-min period. On days 1 and 2 of the test week, mice were acclimatized to the device; on days 3 and 4, average systolic blood pressure data were obtained from 40–60 measurements. Electrocardiograms were obtained, as described previously [22], on un-anesthetized, unrestrained mice using the ECGenie system (Mouse Specifics, Quincy, MA), which consists of three pediatric conductance leads on the platform of a 12-in high tower. Mice were acclimatized for 5–10 minutes and measurements were obtained in the following 3–5 minute period. Whole body areal bone mineral density and percent body fat were measured using the PIXImus dual energy X-ray densitometer (GE-Lunar, Madison, WI) as previously described [22]. Full body scans were obtained and X-ray absorptiometry data were gathered and processed with manufacturer-supplied software (Lunar PIXImus 2,vers. 2.1). The head was specifically excluded from all analyses due to concentrated mineral in skull and teeth. Mice were weighed on a Navigator scale (Ohaus Corporation, Florham Park, NJ) set to the “animal weighing” mode. The adrenal glands, kidneys, heart, and testes were weighed at necropsy.

**Table 2:** Schedule of assays.

	Age of mice at test (weeks)^a^	
Measurement	3-month study	8-month study
Body weight	5, 7, 9, 11, 13	7, 11, 15, 19, 23, 27, 31, 35
Hematological analysis	7	19
Total cholesterol and high-density lipoprotein (HDL)	8	30
Blood pressure	9	29
Electrocardiogram	11	31
Behavioral testing	14	34
Body composition and bone mineral density	15	35
Organ weights	15	35
Behavioral observation	3× weekly	3× weekly

a Mice were weaned and entered into the experimental protocol at 3 weeks of age, except B6 mice, which were weaned and entered at 4 weeks of age, their normal wean age; hence, B6 were 1 week older at the time of each measurement.

Mice were observed three times a week for mortality, morbidity and aggressive behavior (fighting, tail biting), compulsive behavior (whisker-picking, barbering), and stress (alopecia). In addition, behavior was assessed with the open field and light/dark tests using the VersaMax Animal Activity Monitoring System (AccuScan Instruments Inc., Columbus, Ohio). Mice were tested for behavior on the same day of the week between 0800 and 1200 h by the same technicians. The open field test measured activity level, exploratory behavior, and anxiety-like behavior of individual mice in an acrylic arena (40 cm×40 cm; light provided by 7.5-W red light bulb). During a 10-minute period, mice were placed in the center of the open field and monitored for time spent and distance traveled in the center of the arena and in the entire arena. The light–dark test measured anxiety-like behavior using a modified arena that included a dark enclosure (Figure S1). Mice were placed in the dark enclosure and monitored for 10 minutes, to measure the time elapsed before the first move to the light side, the number of changes between light and dark, and the total time spent in the light side. Because these behavioral tests were so time-consuming, only the mice in shoebox cages were measured. Furthermore, several sets of mice from each strain were put through our testing protocols for validation and calibration of the equipment. During these preliminary trials, only 1 of 24 strain 129 mice and 1 of 12 A/J mice ventured into the light side. Based on these results, we discontinued testing of these 2 strains.

### Statistical Analysis

Analysis of whether increased density had any effect was done for each strain separately since it is possible that density might affect some strains more than others. Likewise, males and females were analyzed separately, because sex is known to affect some traits. For most traits, three mice per cage were measured as the technical replicates, regardless of the number of mice in the cage, and the measurements were averaged for a cage mean. Mice to be used as technical replicates were ear marked, and the same three mice were measured each time. For each sex, trait, and group, we used six cages and averaged these cages to provide the biological replicates, reported as mean ± standard error (SEM). The effect of density alone was investigated per phenotype, and if a significant effect was found, the Tukey HSD (post hoc) test was used to determine significant differences between density factor levels. The software for statistical modeling was SAS v9.3 and JMP v10.0.0, both from SAS Institute (Cary, NC), and R v2.15.0 [24].

Hematology, plasma cholesterol, blood pressure, electrocardiogram, body composition and organ weight data were analyzed by linear regression. Body weight was used as a covariate for organ weight. For body weight, repeated measures linear regression models were fit to measurements taken throughout the study. For repeated measures analyses, a compound symmetry covariance matrix was fit to the data to account for the presence of correlation between observations. Firth's bias reduction was used for traits observed in low frequency. Negative binomial regression models with Firth's bias reduction were fit to mortality measurements for the duplex and shoebox cage types in the 3-month study. Firth's bias reduction was applied to the duplex cages only in the 8-month study. Logistic regression models with Firth's bias reduction were fit to data from the behavioral observations and the incidence of alopecia. Behavior testing data were collected from two assays, the open field and light/dark methods for the 8-month timeframe. Negative binomial regression models were fit to the following light/dark data: light side time, side changes, and reaction time. Linear regression models were used for the following open field data: center time and total distance.

## Results

**Figure 1:**
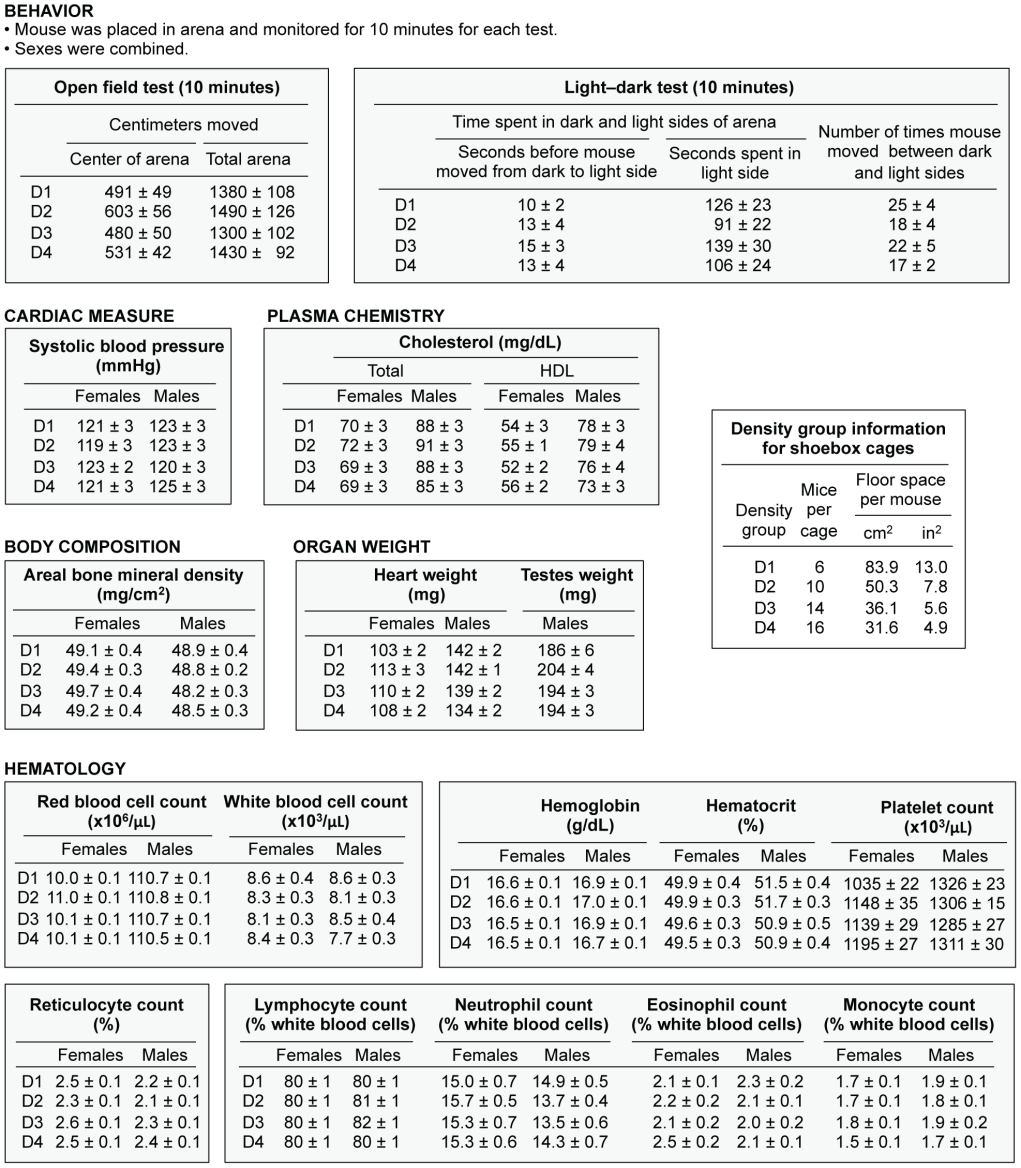
Values of traits for which housing density had no significant effect. Values (mean ± SEM) are shown for BALB in shoebox cages, 3-month timeframe, except that behavior is shown for 8-month timeframe. Values for all strains are in Supporting Information.

Our study evaluated the effects of housing density on 27 traits in five inbred strains. Housing density had no significant effect on 23 traits: body weight, systolic blood pressure, HDL and total plasma cholesterol, areal bone mineral density, heart and testes weight, and hematology (red and white blood cell counts, hemoglobin, hematocrit, platelet count, lymphocytes, neutrophils, reticulocytes, eosinophils, monocytes). Increased housing density significantly reduced kidney weight, adrenal weight, and heart rate. In contrast, percent fat increased as housing density increased but only in specific strains. Complete data are provided for these four traits that showed a significant effect of housing density. Complete data are also provided for mortality, body weight, behavioral traits (fighting and tail biting, whiskering and barbering, and alopecia) and open field and light–dark performance. For the other traits, for which no significant effects of housing density were found, a representative data set is shown in Figure 1 for strain BALB only, due to the extensive amount of data obtained in this study. However, all data for the five strains are shown in supplementary information (Tables S1, S2, S3, S4, S5, S6, S7, S8, S9, S10, S11).

**Figure 2:**
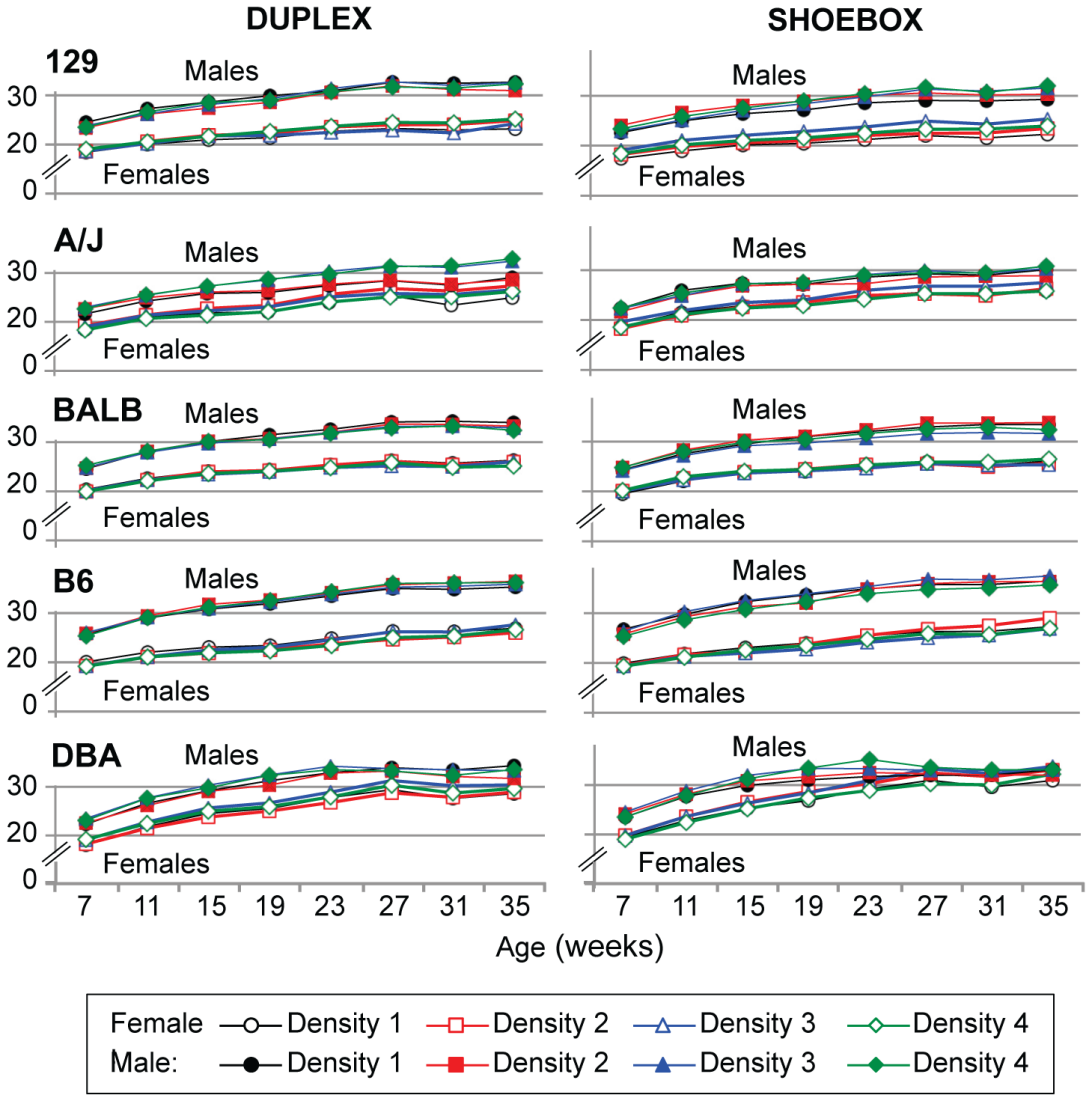
Body weight (g). N = 16–18 animals per sex/strain. Standard errors are not shown because the maximum standard errors are within the size of the symbols.

Most traits did not differ significantly based on the number of mice in a cage. For example, the percent mortality did not differ among the density groups although mortality did differ from strain to strain (Table 3). Mortality rate in the 8-month timeframe varied from 0.1% in B6 mice to 1.8% in strain 129, 3.6% in BALB, 4.3% in A/J, and 6.3% in DBA. Body weight also did not differ as the density of mice increased; however, there was a significant difference between males and females for each strain (Fig. 2 and Table S1). Increasing housing density did not result in a consistent effect on behavioral traits, although these traits did differ from strain to strain; at the 8-month timeframe, fighting and tail biting were observed in 30% of DBA cages, alopecia in 35% of B6 cages, and whiskering and barbering in 91% of strain 129 cages (Table 4). All these behavioral traits were somewhat elevated in strain A/J (fighting and tail biting 10%, whiskering and barbering 11%, alopecia 21%). The open field and light–dark assays, evaluated for the 8-month timeframe, did not show a pattern that could be ascribed to housing density (Table 5 and Figure S2). Two strains, 129 and A/J, showed low activity in the open field test, often remaining in the center where they were placed, and no activity in the light–dark test; these two strains did not move into the lighted arena from the dark enclosure. The remaining three strains, BALB, B6, and DBA, showed no consistent differences that could be ascribed to housing density for center distance, center time, total distance in the open field or light side reaction time, light side changes, or total time spent in the light side.

**Table 3:** Mortality (percentage of mice that died during the study).

		Percentage of mice that died per strain per density group										
	Total mice per density group		129		A/J		BALB		B6		DBA	
Cage type		Density group^a^	3-mo	8-mo	3-mo	8-mo	3-mo	8-mo	3-mo	8-mo	3-mo	8-mo
Duplex	48	1	0	2	0	4	0	13	2	0	0	8
	84	2	0	1	1	1	1	5	0	0	4	11
	108	3	0	3	3	6	1	1	0	0	1	15
	132	4	0	3	2	8	0	1	0	0	0	6
Shoebox	72	1	0	1	0	3	0	3	0	0	0	4
	120	2	0	2	0	3	0	3	0	0	0	0
	168	3	0	1	1	6	0	2	0	0	0	3
	192	4	0	1	1	3	1	1	0	1	0	3
Average			0	1.8	1.0	4.3	0.4	3.6	0.3	0.1	0.6	6.3

Sexes are combined.

a For details of floor space for each density group, see Table 1.

Mortality did not differ significantly among density groups, but it did differ among strains.

**Table 4:** Behavioral observations.

				Number of cages (out of 24) in which behavior was observed at least once							
	Density group^a^	129		A/J		BALB		B6		DBA	
		3-mo	8-mo	3-mo	8-mo	3-mo	8-mo	3-mo	8-mo	3-mo	8-mo
Fighting, tail biting	1	0	0	0	3	2	0	0	0	0	2
	2	0	0	0	1	0	1	0	0	0	9
	3	0	0	0	3	2	2	0	1	0	13
	4	0	0	2	3	1	1	0	2	0	5
Whiskering, barbering	1	11	20	0	2	0	0	1	1	0	1
	2	14	22	0	0	0	2	0	3	0	0
	3	14	23	0	2	0	3	0	2	0	0
	4	14	22	0	7	0	1	1	1	0	0
Alopecia	1	0	0	0	1	0	0	2	5	0	0
	2	0	0	0	5	0	0	4	8	0	0
	3	0	0	0	7	0	1	2	10	0	0
	4	0	0	0	7	0	0	2	11	0	0

Sexes and cage types are combined, for a total of 24 cages per strain/timeframe/density (2 sexes ×2 cage types ×6 replicates = 24 cages). The value represents the number of cages of the 24 in which the behavior was observed at least once during the entire 3-month or 8-month timeframe. For example, for strain 129, density group 1, whiskering and barbering were observed in 11 of the 24 cages during the 3-month timeframe and in 20 of the 24 cages during the 8-month timeframe. Alopecia was not observed in any of the strain 129 or DBA cages, regardless of density group or timeframe.

a For details of floor space for each density group, see Table 1.

Behavioral observations did not differ significantly among density groups. Although behavioral observations did differ among strains, all values were within normal ranges.

**Table 5:** Open field and light–dark activity within each 10-minute test for 8-month timeframe, shoebox cages.

		Open field tests			Light-dark tests		
		Distance moved (cm)			Amount of time (sec)		
Strain	Density group^a^	In center of arena	In total arena	Percent time in center of arena (%)	Prior to move from dark	In light	Moves between dark and light
129S1/SvImJ	1	30±7	107±24	24±31	—	—	—
	2	22±4	66±8	32±35	—	—	—
	3	35±5	99±15	32±28	—	—	—
	4	33±6	29±24	27±32	—	—	—
A/J	1	42±7	165±19	18±26	—	—	—
	2	40±8	137±19	24±31	—	—	—
	3	31±5	91±7	21±27	—	—	—
	4	36±6	105±9	21±29	—	—	—
BALB/cByJ	1	491±49	1380±108	21±14	10±2	126±23	25±4
	2	603±56	1490±126	26±15	13±4	91±22	18±4
	3	480±50	1300±102	23±18	15±3	139±30	22±5
	4	531±42	1430±92	22±11	13±4	106±24	17±2
C57BL/6J	1	146±21	790±67	7±6	67±25	56±17	5±1
	2	184±53	842±158	8±6	93±26	92±28	5±1
	3	126±21	725±69	9±14	102±27	48±18	4±1
	4	217±25	1050±88	8±6	73±23	125±24	8±1
DBA/2J	1	289±32	995±88	23±19	117±23	104±21	18±2
	2	256±35	785±88	29±24	147±29	58±20	9±2
	3	308±37	855±76	30±23	158±29	49±10	15±2
	4	245±30	704±65	35±25	96±24	82±26	9±2

Values = mean ± SEM. Sexes were combined (N = 33–36).

Individual mice were placed in the arena and monitored for 10 minutes for each test.

Duplex cages were not tested. Strains 129/S1/SvImJ and A/J are not included in light–dark testing because these mice did not venture from the dark enclosure.

a For details of floor space for each density group, see Table 1.

Behavioral observations did not differ significantly among density groups, but they did differ among strains.

Blood pressure was not affected as housing density increased (Table S3), and neither were HDL and total cholesterol levels (Table S5). Bone mineral density, likewise, did not differ significantly as the housing density increased (Table S4). A group of traits, measured at the termination of the experiment, showed no significant difference in values that could be attributed to the housing density, including heart weight (Table S6), testes weight (Table S6), and all hematological parameters (Table S7, S8, S9, S10, S11). Thus, density did not affect most traits measured.

This study was done in two different cage types. Because the density was kept the same, this meant that the larger shoebox cages had more mice per group (Table 1). Results were similar in both the shoebox and duplex cage types; thus, the study carried out in shoebox serves as a replication of duplex cages.

### Density significantly affected kidney weight, adrenal weight and heart rate

**Figure 3:**
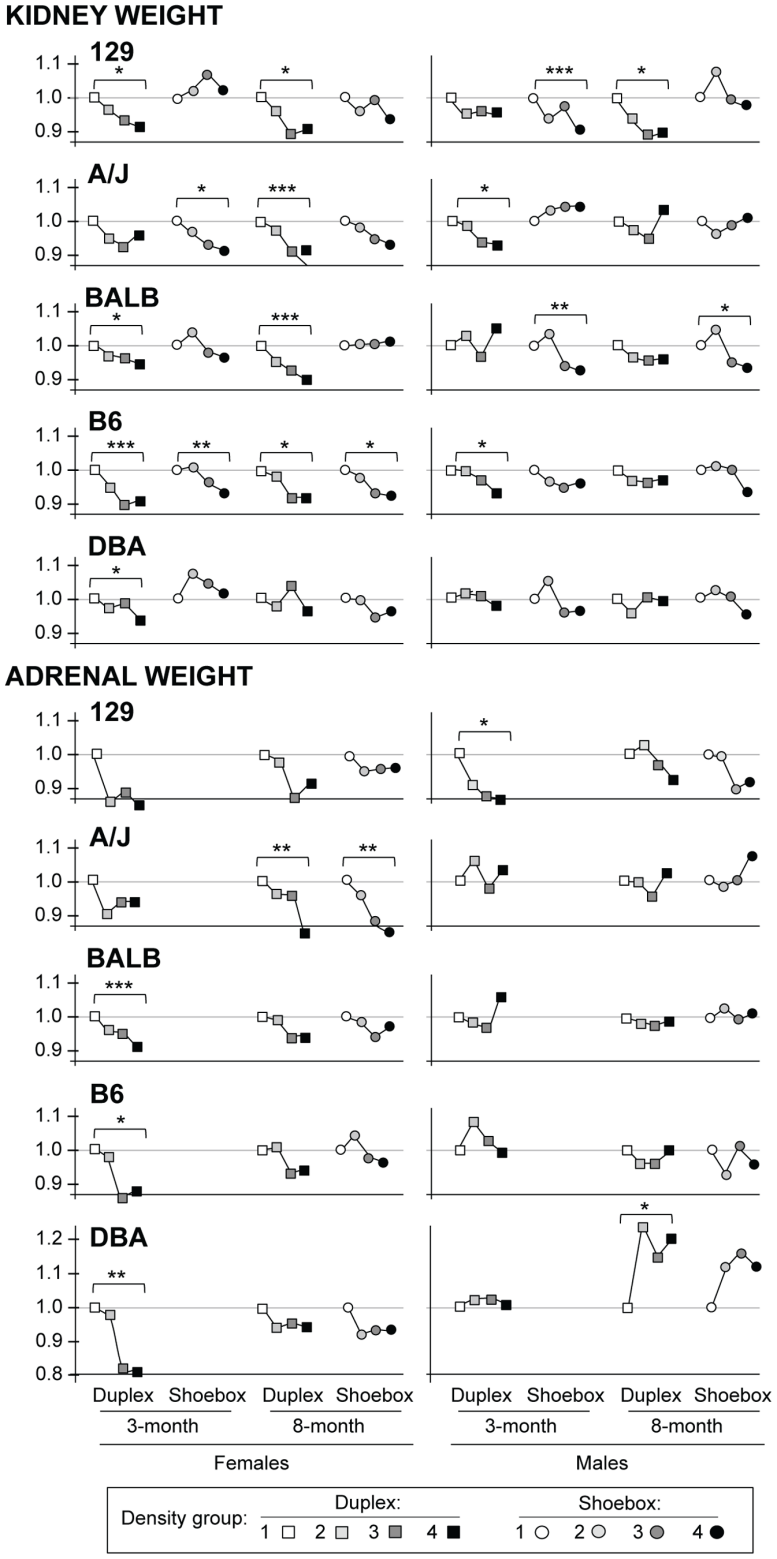
Kidney weight and adrenal weight. N = 16–18 animals per sex/strain. The lowest density is given a value of 1.0 and all other densities are relative to that. Mean values and SEM are shown in Tables S2 and S3. The 3-month timeframe, shoebox, adrenal weight was not measured. *P*-values are comparisons of Density group 1 vs. Density group 4 only: *P<0.05; **P<0.01; ***P<0.005.

**Figure 4:**
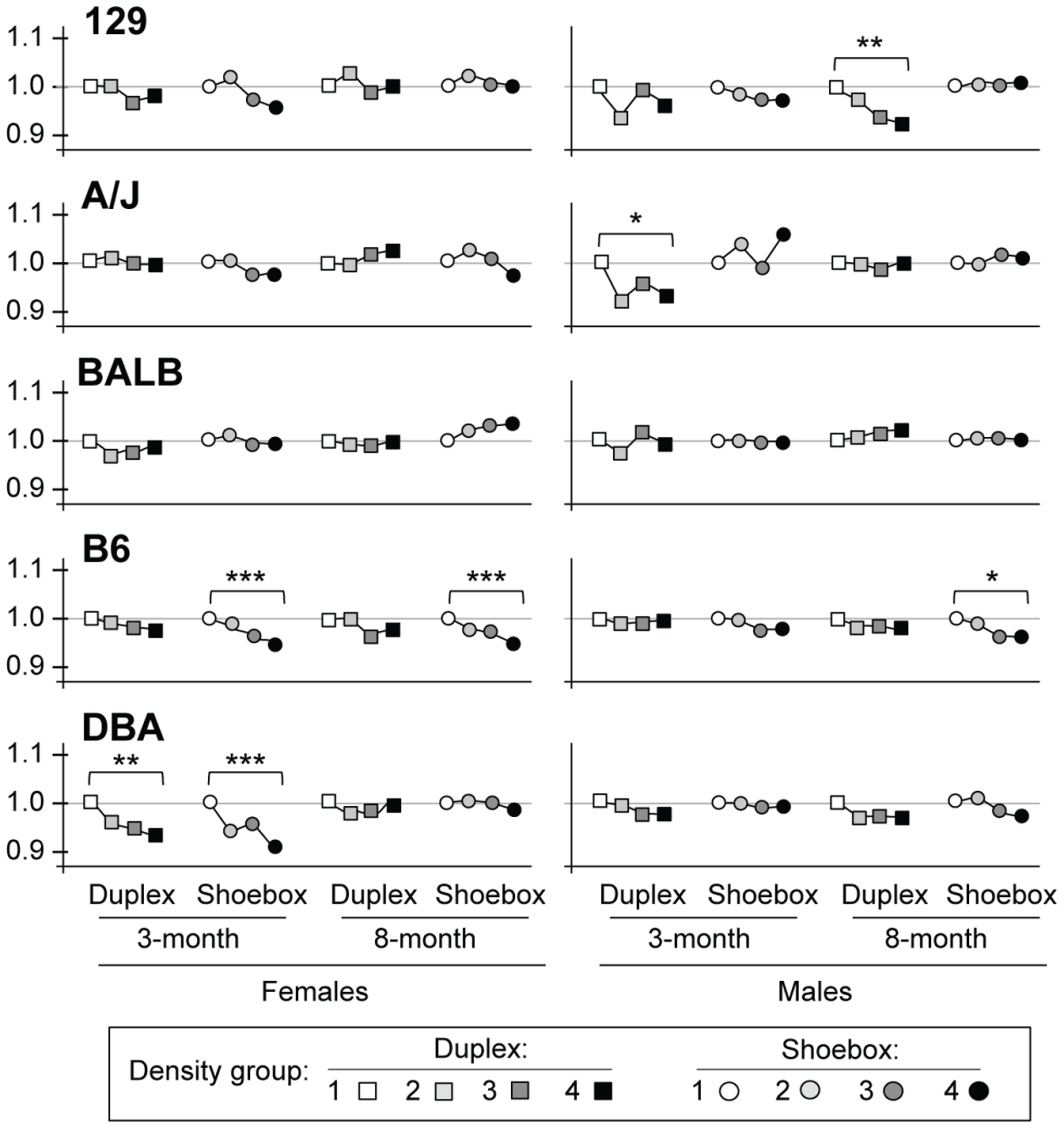
Heart weight. N = 16–18 animals per sex/strain. The lowest density is given a value of 1.0 and all other densities are relative to that. Mean values and SEM are shown in Tables S2 and S3). *P*-values are comparisons of Density group 1 vs. Density group 4 only: *P<0.05; **P<0.01; ***P<0.005.

Kidney weight, adrenal weight, and heart rate were significantly affected by housing density; values were reduced as housing density increased (Fig. 3 and 4); however, values for all traits were within normal range. Those values for which the differences were statistically different between density groups 1 and 4 are depicted with three P-values: *** is P<0.0005, ** is P<0.005, * is P<0.05. To more readily compare the changes caused by housing density, the values in Figures 3 and 4 are depicted as relative to the lowest density, which is given a value of 1.0. Mean values and SEM are shown in Tables S2 and S3).

Kidney weight, the trait most affected by housing density, decreased as the housing density increased (Fig. 3, Table S2). Females were more likely to show a significant decrease than males; female B6 mice showed the most consistent decrease, and strain DBA was the least affected by differences in housing density. Even though kidney weight was the trait showing the greatest decrease with density, all values were within normal range. The amount of decrease was minimal compared to the differences in kidney weight between males and females. For example, for B6 females, the kidney weights were between 233±4 mg and 211±4 mg (a difference of 22 mg) for the lowest and highest densities. However, the value for males at the lowest density was 268±6 mg, a value exceeding the range for females. The 35-mg difference between females (233±4 mg) and males (268±6 mg) of the same strain is greater than the differences that correlate with density.

Adrenal weight also decreased as the housing density increased (Fig. 3, Table S2); data were not collected for the 3-month timeframe, shoebox cages. The decrease was greatest in strain 129, but only the males in the 3-month timeframe reached statistical significance (*P*<0.05). The males in strains A/J, BALB, B6, and DBA did not show decreases in adrenal weight. However, most females did show a decrease of adrenal weight as density increased; this decrease reached significance in duplex cages for strains BALB, B6 and DBA and in shoebox cages for strain A/J.

Heart rate also decreased as housing density increased (Fig. 4, Table S3); however, the pattern was not as consistent as the patterns for kidney and adrenal weights. Moreover, some strains showed no decrease (both sexes in strain BALB; females in 129 and A/J; males in DBA). Only a few of the decreases reached statistical significance; B6 females in shoebox cages and DBA females in both cage types in the 3-month timeframe.

### Density significantly affected percent fat only in specific strains

**Figure 5:**
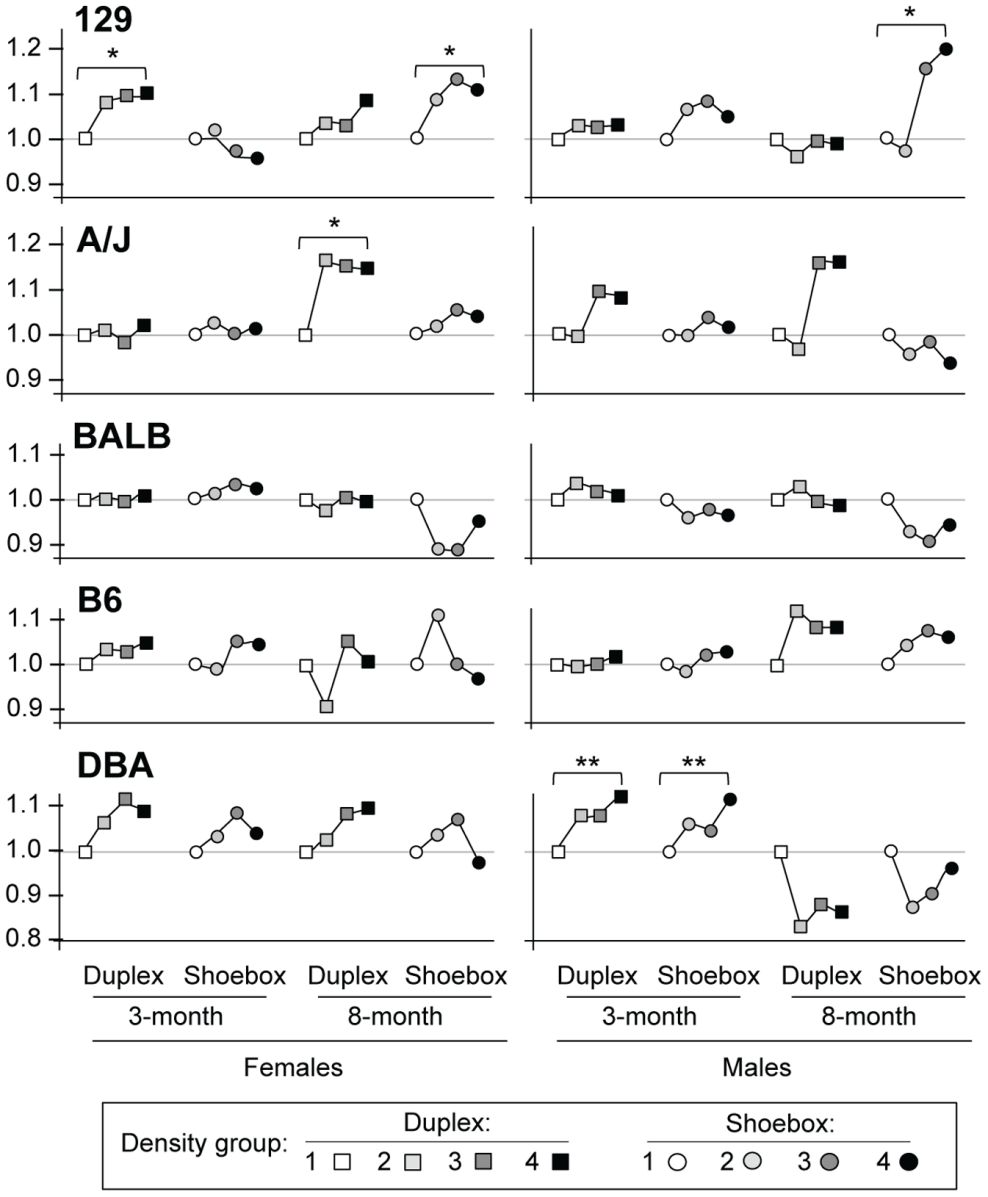
Percent fat. N = 16–18 animals per sex/strain. The lowest density is given a value of 1.0 and all other densities are relative to that. Mean values and SEM are shown in Tables S2 and S3). *P*-values are comparisons of Density group 1 vs. Density group 4 only: *P<0.05; **P<0.01; ***P<0.005.

The previous three traits (kidney weight, adrenal weight, heart rate) decreased as housing density increased. In contrast, percent body fat increased as housing density increased. The increased fat was most pronounced in the 129 strain and was not observed in BALB (Fig. 5, Table S4).

## Discussion

This study tested whether mice housed more densely, approximately 2, 2.6, and 3 times greater than recommended by the *Guide*, showed any differences in measureable parameters describing health and well-being. Increased housing density had little effect on 23 of 27 traits measured. Density consistently affected kidney weight, adrenal weight and heart rate. Values for these traits were reduced as housing density increased. Density also affected percent body fat, but only in certain strains, where increased density resulted in increased fat. Measurements for all four of these traits that differed were within physiologically normal ranges: kidney weight [25], adrenal weight [26], heart rate [27], percent body fat [23]. The difference between the values at the lowest and highest densities was less than the difference between females and males of the same strain. An increase in body fat may be the result of increased cage temperature that is closer to the thermoneutral zone for mice. However, all changes were small compared to normal variation among different strains.

Kidney weight showed the most consistent pattern, with decreased organ weight as housing density increased across both timeframes and cage types and for all five strains. Adrenal weight is often used as a measure of stress, reflecting degree of chronic stress hormone production [28]–[30], and the trend in decreased adrenal weight we observed suggests that mice housed more densely were less stressed; however we recognize that changes did not reach statistical significance. Heart rate, another commonly used measure of stress [31]–[33], decreased as density increased. This observation may indicate that these mice were less stressed. Our study is consistent with two previous studies in mice, which demonstrated a reduction in heart rate with increased housing density [8], [34]. Previous studies used the invasive technique of surgically implanting telemeters, but our study measured heart rate non-invasively in mice removed from their cage for a conscious electrocardiogram test. Thus, the reduced heart rate not only applies within the cage, but also is maintained when mice are temporarily removed from the cage and placed in a novel situation. The decreased heart rate appears to be a positive effect of increased housing density.

In contrast, percent body fat increased for mice in duplex cages for both timeframes, with the exception of DBA males at the 8 month time point. We suggest that this increased body fat is a result of increased heat conservation. As mice are housed more densely, they require fewer calories to maintain body temperature and thus can store some as fat [35], [36]. Whether this indicates anything about the well-being of mice is open to interpretation.

### Issues with multiple testing

One challenge in analyzing such a large number of traits was defining the appropriate significance level. We corrected for multiple “within-trait” comparisons of cage type, density, and sex with the Tukey HSD *post hoc* analysis, but we chose not to correct for inclusion of 27 different traits, some of which are related, particularly hematological traits. Imposing the commonly used Bonferroni correction for multiple comparisons would have resulted in a statistical significance threshold of *P* = 0.002 (the traditional 0.05÷27 parameters). Because the purpose of this study was to find any sign of an adverse effect, a *P*-value this rigorous could have hidden legitimate findings. Therefore, we reported all *P* values 0.05 or lower, allowing readers to evaluate appropriate significance.

### Comparison of these results with other studies

Our results concur with many previous studies [2], [3], [7], [8], [11], [12], [14], [35]–[43] indicating that many strains of mice can be housed at twice the *Guide's* density recommendation. Many results from previous studies suggest positive health benefits for more densely housed mice, including enhanced immune response [3], [7] and behavior that indicated reduced anxiety [2], [7].

### Limitations of this study

We used inbred strains in our study. It will be valuable to replicate our results in hybrid or outbred stocks, as well as in other inbred strains with inherently different body size or metabolic rate. Our study was carried out in individually ventilated caging, which ensures acceptable air quality at all densities we tested. In conventional caging systems that depend on room ventilation, increased housing density might produce negative effects in mice due to a reduction in cage air quality. Other density studies measured immune function, which was not included in our experimental design, such as plasma corticosterone [3], [6], [41], NK cell activity [3], and splenic T-cell subpopulations [6]. Lastly, it is difficult to draw conclusions from the absence of adverse effects because there might have been adverse effects that we did not measure.

We wanted to include cage types widely used by the scientific community. Therefore, we used the two most commonly used cages in parallel and simply varied the number of mice per cage, which changed both density and the total number of interacting mice. Some studies kept the number of mice consistent and increased density by reducing the size of the cage [2], [3], [7]; their conclusions did not differ from ours.

### Conclusion

In conclusion, these data indicate that mice can be housed at greater densities than those commonly used. The latest *Guide* does allow greater densities per cage if this is supported and if these greater densities have been tested and found to be acceptable. In our study, most traits were not affected by increased housing density, and three traits that were affected may indicate that increased housing density results in less stress for mice.

## Supporting Information

Figure S1 **LightDarkArena131029.** Modified enclosure for the light-dark test.
(TIF)

Figure S2 **Activity140107.** Representative samples of locomotor activity in the open field for each of the five strains.
(PDF)

Table S1 **Body Weight 131029.** Table S1A: Weight of mice (g) at specific ages during the 3-month timeframe. Table S1B: Weight of mice (g) at specific ages during the 8-month timeframe.
(XLSX)

Table S2 **Kidney,AdrenalWt121029.** Kidney and adrenal weight (mg) for each of 5 strains for both the 3-month and 8-month timeframes.
(PDF)

Table S3 **HeartRate, BloodPres131029.** Heart rate (bpm) and blood pressure (mmHg) for each of 5 strains for both the 3-month and 8-month timeframes.
(PDF)

Table S4 **PercentFat,BMD131029.** Percent fat (%) and areal bone mineral density (mg/cm^2^) for each of 5 strains for both the 3-month and 8-month timeframes.
(PDF)

Table S5 **Cholesterol131029.** HDL and total cholesterol (mg/dL) for each of 5 strains for both the 3-month and 8-month timeframes.
(PDF)

Table S6 **Heart, TestesWt131029.** Heart weight and testes weight (mg) for each of 5 strains for both the 3-month and 8-month timeframes.
(PDF)

Table S7 **RBC,WBC121029.** Red (×10^6^/µL) and while blood cell counts (×10^3^/µL) for each of 5 strains for both the 3-month and 8-month timeframes.
(PDF)

Table S8 **Hemoglobin, Heatrocrit131029.** Hemoglobin (g/dL) and hematocrit (% red blood cells/total volume of blood) for each of 5 strains for both the 3-month and 8-month timeframes.
(PDF)

Table S9 **Platelets,Lymphocytes131029.** Platelet count (×10^3^/µL) and lymphocyte count (% white blood cells) for each of 5 strains for both the 3-month and 8-month timeframes.
(PDF)

Table S10 **Neutro,Reticulocyes131029.** Neutrophil count (% white blood cells) and reticulocyte count (%) for each of 5 strains for both the 3-month and 8-month timeframes.
(PDF)

Table S11 **Eosinophils,Monocytes131029.** Eosinophil count and monocyte count (% white blood cells) for each of 5 strains for both the 3-month and 8-month timeframes.
(PDF)
