# Supplementary figures and images for: Effects of Housing Density in Five Inbred Strains of Mice
Source: PLoS One. 2014 Mar 21;9(3):e90012. doi: 10.1371/journal.pone.0090012 (PMC3962340; doi:10.1371/journal.pone.0090012)

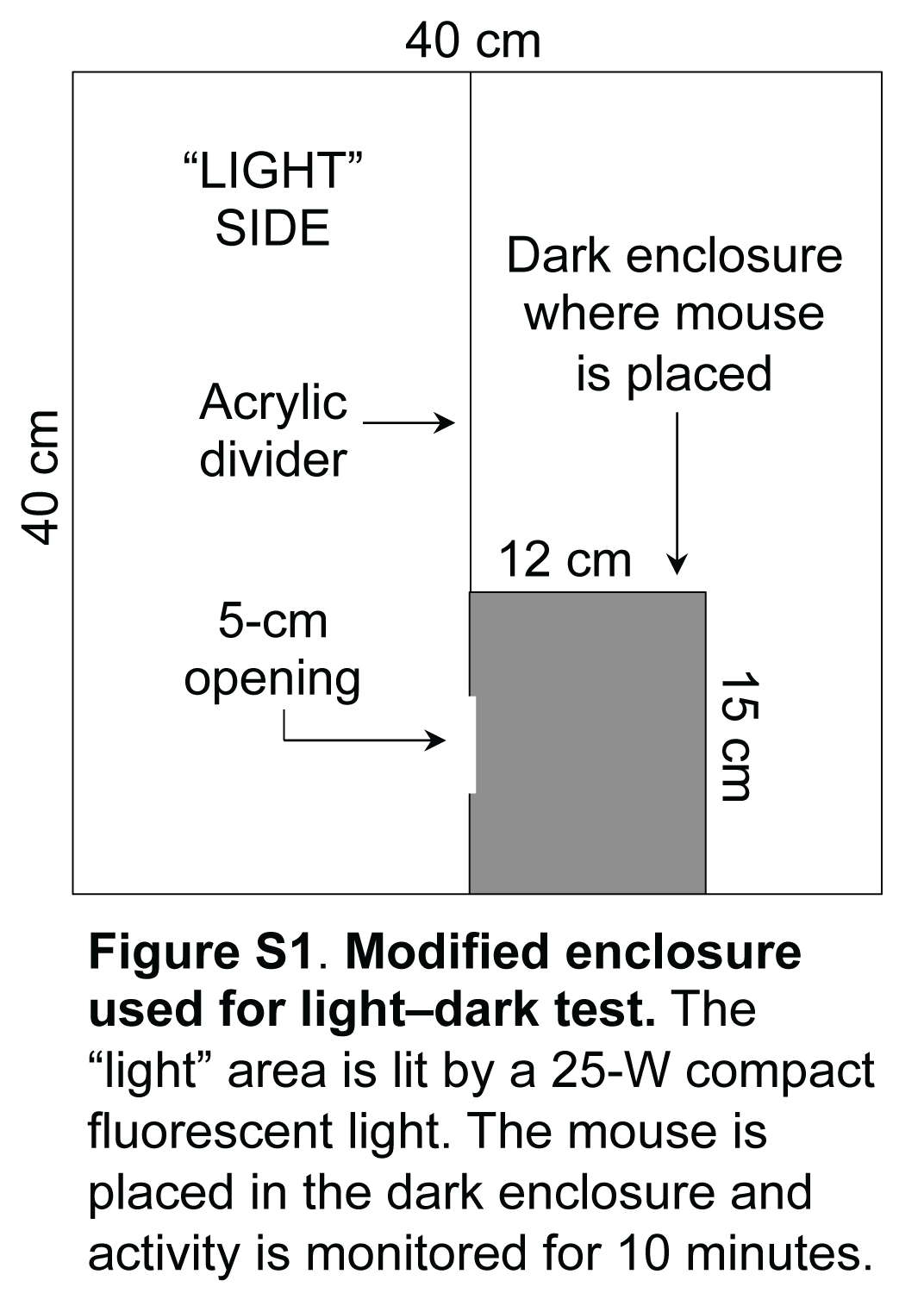

Supplement: Figure S1 — LightDarkArena131029. Modified enclosure for the light-dark test. (TIF) [file pone.0090012.s001.tif]
