# Supplementary material for: Effects of Housing Density in Five Inbred Strains of Mice
Source: PLoS One. 2014 Mar 21;9(3):e90012. doi: 10.1371/journal.pone.0090012 (PMC3962340; doi:10.1371/journal.pone.0090012)
Supplement: Figure S2 — Activity140107. Representative samples of locomotor activity in the open field for each of the five strains. (PDF) [file pone.0090012.s002.pdf]

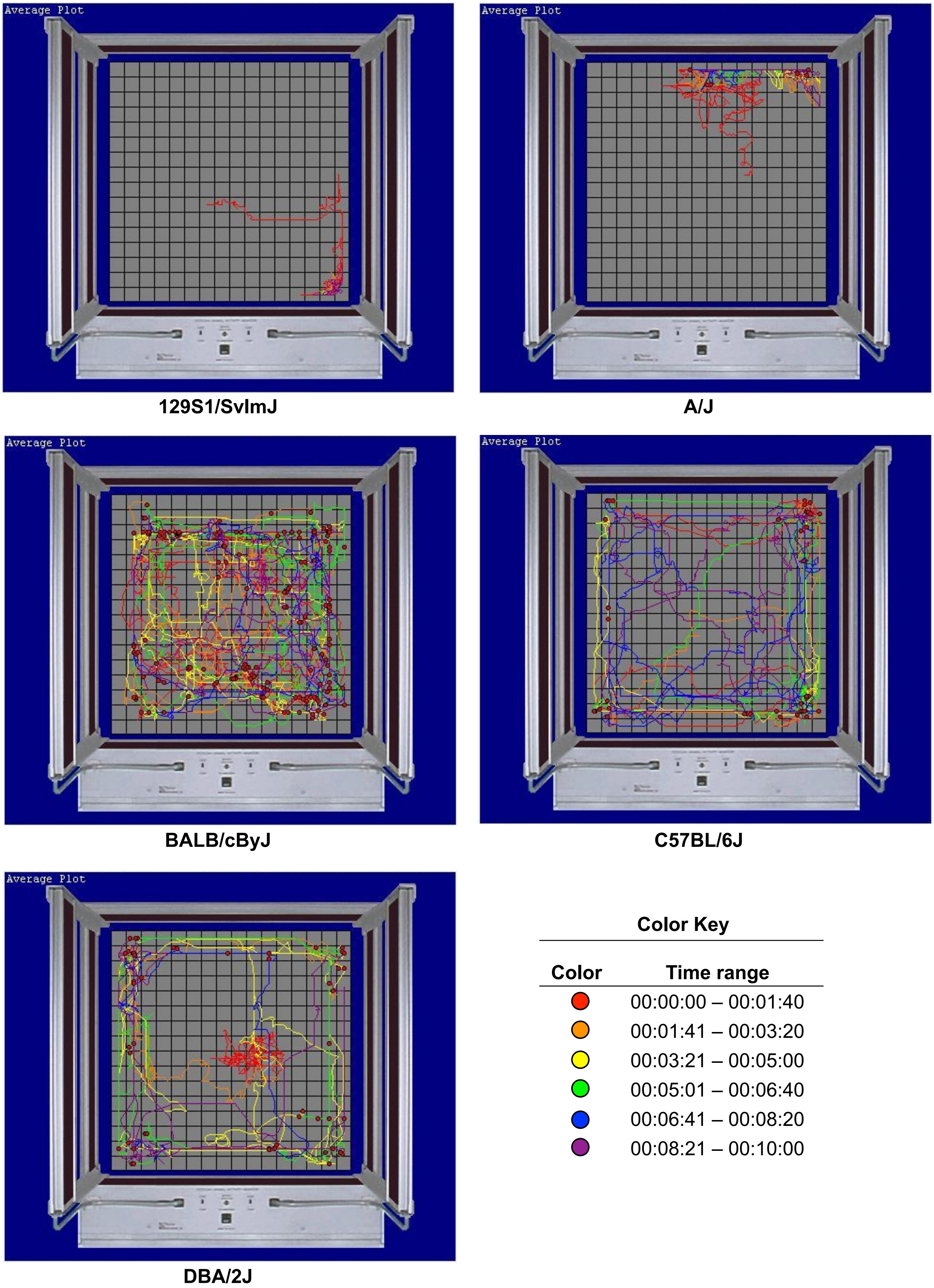

Figure S2. Representative samples of locomotor activity in the open field for each of the five strains (Accuscan VersaPlot software image).
