# Supplementary material for: Effects of Housing Density in Five Inbred Strains of Mice
Source: PLoS One. 2014 Mar 21;9(3):e90012. doi: 10.1371/journal.pone.0090012 (PMC3962340; doi:10.1371/journal.pone.0090012)
Supplement: Table S2 — Kidney,AdrenalWt121029. Kidney and adrenal weight (mg) for each of 5 strains for both the 3-month and 8-month timeframes. (PDF) [file pone.0090012.s004.pdf]

Table S2. Kidney weight and adrenal weight.

Time-frame	Density group[a]	129S1/SvImJ		A/J		BALB/cByJ		C57BL/6J		DBA/2J	
		Duplex	Shoebox	Duplex	Shoebox	Duplex	Shoebox	Duplex	Shoebox	Duplex	Shoebox
**KIDNEY WEIGHT (mg)**											
**Females**											
3-month	1	228 ± 7	194 ± 4	243 ± 4	250 ± 6	275 ± 5	263 ± 8	233 ± 4	222 ± 3	237 ± 5	230 ± 6
	2	220 ± 5	199 ± 6	231 ± 5	243 ± 6	267 ± 4	273 ± 6	220 ± 3	223 ± 4	231 ± 3	246 ± 5
	3	212 ± 7	208 ± 5	225 ± 6	233 ± 5	265 ± 4	258 ± 5	208 ± 4	213 ± 4	233 ± 5	240 ± 6
	4	208 ± 5*	200 ± 5	233 ± 5	229 ± 6*	259 ± 5*	253 ± 5	211 ± 4***	207 ± 3**	222 ± 5*	233 ± 6
8-month	1	253 ± 6	233 ± 6	289 ± 8	272 ± 7	293 ± 5	283 ± 3	268 ± 6	280 ± 4	277 ± 7	285 ± 5
	2	244 ± 5	224 ± 9	281 ± 7	268 ± 9	279 ± 4	284 ± 5	263 ± 6	274 ± 5	271 ± 6	284 ± 7
	3	227 ± 10	232 ± 9	263 ± 6	258 ± 5	271 ± 5	284 ± 5	246 ± 7	261 ± 6	286 ± 7	270 ± 5
	4	231 ± 6*	218 ± 4	251 ± 7***	254 ± 8	263 ± 4***	286 ± 5	246 ± 5*	259 ± 6*	266 ± 5	274 ± 6
**Males**											
3-month	1	354 ± 13	320 ± 10	283 ± 7	267 ± 6	417 ± 10	438 ± 8	310 ± 6	301 ± 8	361 ± 6	366 ± 9
	2	336 ± 9	300 ± 11	278 ± 8	276 ± 6	429 ± 7	453 ± 9	309 ± 9	291 ± 6	366 ± 8	385 ± 8
	3	340 ± 8	311 ± 8	264 ± 4	279 ± 8	404 ± 8	412 ± 9	300 ± 7	285 ± 6	363 ± 10	350 ± 9
	4	338 ± 10	290 ± 7***	262 ± 5*	278 ± 6	438 ± 9	405 ± 7**	287 ± 7*	289 ± 7	352 ± 9	352 ± 9
8-month	1	402 ± 10	356 ± 10	309 ± 7	305 ± 7	543 ± 8	516 ± 8	336 ± 5	340 ± 5	474 ± 12	483 ± 9
	2	376 ± 13	384 ± 11	301 ± 7	294 ± 9	524 ± 7	540 ± 9	325 ± 6	345 ± 8	453 ± 10	493 ± 10
	3	356 ± 12	354 ± 8	293 ± 7	302 ± 6	520 ± 10	489 ± 11	324 ± 5	341 ± 8	476 ± 16	484 ± 9
	4	360 ± 9*	348 ± 11	319 ± 7	309 ± 10	522 ± 11	481 ± 11*	326 ± 5	317 ± 8	471 ± 16	459 ± 10
**ADRENAL WEIGHT (mg)**											
**Females**											
3-month	1	4.7 ± 0.2	—	6.4 ± 0.3	—	7.1 ± 0.1	—	5.9 ± 0.2	—	5.9 ± 0.3	—
	2	4.1 ± 0.2	—	5.8 ± 0.2	—	6.8 ± 0.1	—	5.7 ± 0.2	—	5.7 ± 0.2	—
	3	4.2 ± 0.2	—	6.0 ± 0.3	—	6.7 ± 0.1	—	5.0 ± 0.1	—	4.7 ± 0.2	—
	4	4.0 ± 0.2	—	6.1 ± 0.2	—	6.5 ± 0.1***	—	5.2 ± 0.2*	—	4.6 ± 0.3**	—
8-month	1	4.4 ± 0.2	4.0 ± 0.1	5.9 ± 0.2	5.9 ± 0.3	6.7 ± 0.1	6.9 ± 0.2	5.8 ± 0.1	5.9 ± 0.2	5.1 ± 0.1	5.1 ± 0.2
	2	4.3 ± 0.1	3.8 ± 0.1	5.7 ± 0.3	5.7 ± 0.2	6.6 ± 0.1	6.8 ± 0.2	5.8 ± 0.2	6.1 ± 0.1	4.7 ± 0.2	4.7 ± 0.1
	3	3.9 ± 0.1	3.8 ± 0.1	5.7 ± 0.2	5.2 ± 0.1	6.3 ± 0.1	6.5 ± 0.2	5.4 ± 0.2	5.7 ± 0.2	4.8 ± 0.2	4.7 ± 0.1
	4	4.1 ± 0.1	3.8 ± 0.1	5.0 ± 0.2**	5.1 ± 0.2**	6.4 ± 0.1	6.8 ± 0.2	5.5 ± 0.2	5.7 ± 0.1	4.7 ± 0.2	4.7 ± 0.2
**Males**											
3-month	1	3.3 ± 0.1	—	2.8 ± 0.1	—	4.2 ± 0.1	—	3.6 ± 0.1	—	2.9 ± 0.1	—
	2	3.0 ± 0.1	—	3.0 ± 0.2	—	4.1 ± 0.1	—	3.9 ± 0.2	—	2.9 ± 0.1	—
	3	2.9 ± 0.1	—	2.7 ± 0.1	—	4.1 ± 0.1	—	3.7 ± 0.2	—	2.9 ± 0.1	—
	4	2.8 ± 0.1*	—	2.9 ± 0.2	—	4.5 ± 0.1	—	3.6 ± 0.2	—	2.9 ± 0.1	—
8-month	1	3.4 ± 0.1	3.4 ± 0.1	3.1 ± 0.1	3.0 ± 0.1	4.4 ± 0.1	4.3 ± 0.1	3.0 ± 0.1	3.2 ± 0.2	3.3 ± 0.1	3.7 ± 0.2
	2	3.5 ± 0.1	3.4 ± 0.1	3.1 ± 0.1	3.0 ± 0.1	4.3 ± 0.1	4.4 ± 0.1	2.9 ± 0.1	3.0 ± 0.1	4.1 ± 0.2	4.1 ± 0.1
	3	3.3 ± 0.1	3.0 ± 0.1	3.0 ± 0.1	3.0 ± 0.1	4.3 ± 0.1	4.3 ± 0.1	2.9 ± 0.1	3.3 ± 0.1	3.8 ± 0.2	4.3 ± 0.2
	4	3.1 ± 0.1	3.1 ± 0.1	3.2 ± 0.1	3.2 ± 0.1	4.4 ± 0.1	4.4 ± 0.1	3.0 ± 0.1	3.1 ± 0.1	4.0 ± 0.2*	4.1 ± 0.2

All values = mean ± SEM. —, not done.
N = 16–18 for each strain/sex/cage/density group.
[a] For details of floor space for each density group, see Table 1.
All *P*-values compare Density 1 : Density 4: **P* < 0.05; ***P* < 0.005; ****P* < 0.0005
