# Supplementary material for: Effects of Housing Density in Five Inbred Strains of Mice
Source: PLoS One. 2014 Mar 21;9(3):e90012. doi: 10.1371/journal.pone.0090012 (PMC3962340; doi:10.1371/journal.pone.0090012)
Supplement: Table S3 — HeartRate, BloodPres131029. Heart rate (bpm) and blood pressure (mmHg) for each of 5 strains for both the 3-month and 8-month timeframes. (PDF) [file pone.0090012.s005.pdf]

Table S3. Heart rate and blood pressure.

Time-frame	Density group[a]	129S1/SvImJ		A/J		BALB/cByJ		C57BL/6J		DBA/2J	
		Duplex	Shoebox	Duplex	Shoebox	Duplex	Shoebox	Duplex	Shoebox	Duplex	Shoebox
**HEART RATE (beats per minute)**											
**Females**											
3-month	1	686 ± 13	711 ± 15	708 ± 20	739 ± 15	701 ± 6	687 ± 7	757 ± 4	748 ± 4	740 ± 8	739 ± 8
	2	689 ± 15	724 ± 13	711 ± 15	738 ± 17	680 ± 6	692 ± 6	748 ± 6	738 ± 5	712 ± 9	696 ± 13
	3	665 ± 17	692 ± 11	704 ± 19	717 ± 15	684 ± 8	682 ± 4	743 ± 5	723 ± 6	702 ± 9	708 ± 6
	4	672 ± 13	681 ± 11	702 ± 17	718 ± 10	690 ± 4	680 ± 7	739 ± 4	709 ± 7***	688 ± 11**	671 ± 9***
8-month	1	654 ± 15	650 ± 9	713 ± 14	700 ± 16	674 ± 9	663 ± 10	734 ± 5	731 ± 5	729 ± 5	714 ± 5
	2	673 ± 14	667 ± 7	706 ± 15	713 ± 13	669 ± 8	676 ± 9	736 ± 5	716 ± 6	715 ± 4	717 ± 4
	3	647 ± 13	655 ± 6	722 ± 13	702 ± 13	667 ± 7	682 ± 6	710 ± 6	712 ± 5	719 ± 6	714 ± 4
	4	655 ± 9	653 ± 10	726 ± 15	677 ± 16	671 ± 6	685 ± 6	720 ± 5	695 ± 5***	726 ± 7	705 ± 5
**Males**											
3-month	1	742 ± 13	746 ± 10	805 ± 10	727 ± 18	700 ± 10	704 ± 7	760 ± 5	752 ± 8	758 ± 10	759 ± 8
	2	696 ± 17	735 ± 9	740 ± 19	754 ± 16	680 ± 14	702 ± 7	752 ± 6	752 ± 5	756 ± 8	758 ± 6
	3	737 ± 10	726 ± 11	767 ± 13	719 ± 17	710 ± 7	702 ± 9	753 ± 5	737 ± 5	740 ± 10	751 ± 5
	4	712 ± 16	726 ± 8	748 ± 13*	770 ± 11	693 ± 7	702 ± 9	759 ± 4	737 ± 4	735 ± 10	754 ± 6
8-month	1	750 ± 9	706 ± 10	729 ± 14	727 ± 11	679 ± 13	707 ± 8	752 ± 3	754 ± 7	753 ± 7	751 ± 8
	2	729 ± 10	710 ± 11	724 ± 17	725 ± 13	684 ± 8	708 ± 9	741 ± 5	748 ± 6	731 ± 12	760 ± 9
	3	703 ± 13	709 ± 10	719 ± 15	738 ± 9	689 ± 13	709 ± 8	742 ± 4	728 ± 5	733 ± 7	740 ± 8
	4	691 ± 12**	714 ± 9	729 ± 11	730 ± 12	694 ± 11	707 ± 5	737 ± 6	726 ± 4*	731 ± 9	730 ± 9
**BLOOD PRESSURE (mmHg)**											
**Females**											
3-month	1	126 ± 6	130 ± 5	116 ± 4	122 ± 4	117 ± 4	121 ± 3	134 ± 3	122 ± 3	127 ± 4	117 ± 2
	2	140 ± 5	135 ± 6	122 ± 5	127 ± 4	113 ± 3	120 ± 3	130 ± 3	123 ± 3	119 ± 4	120 ± 3
	3	131 ± 6	134 ± 5	124 ± 4	123 ± 5	121 ± 4	123 ± 2	133 ± 4	116 ± 2	118 ± 4	118 ± 3
	4	136 ± 7	135 ± 6	122 ± 5	128 ± 3	117 ± 3	121 ± 3	135 ± 3	121 ± 3	119 ± 4	125 ± 4
8-month	1	130 ± 5	123 ± 5	109 ± 6	121 ± 5	113 ± 5	115 ± 2	124 ± 2	113 ± 2	118 ± 3	117 ± 4
	2	125 ± 5	125 ± 5	113 ± 3	116 ± 3	115 ± 4	120 ± 2	128 ± 4	112 ± 3	111 ± 3	111 ± 3
	3	123 ± 5	117 ± 4	114 ± 3	111 ± 4	112 ± 4	116 ± 4	126 ± 3	113 ± 3	115 ± 3	110 ± 3
	4	116 ± 4	124 ± 4	117 ± 3	108 ± 4	113 ± 4	121 ± 3	121 ± 4	118 ± 3	115 ± 3	113 ± 4
**Males**											
3-month	1	135 ± 6	129 ± 4	121 ± 3	121 ± 3	123 ± 2	123 ± 3	116 ± 2	121 ± 2	124 ± 4	118 ± 4
	2	134 ± 7	132 ± 4	119 ± 3	125 ± 3	128 ± 3	123 ± 3	119 ± 2	124 ± 2	124 ± 3	115 ± 4
	3	133 ± 6	131 ± 4	121 ± 3	119 ± 3	121 ± 4	120 ± 3	119 ± 3	119 ± 3	124 ± 4	119 ± 4
	4	135 ± 8	131 ± 4	119 ± 3	124 ± 3	120 ± 3	125 ± 3	117 ± 2	126 ± 3	124 ± 3	124 ± 4
8-month	1	127 ± 4	130 ± 4	112 ± 3	113 ± 5	127 ± 3	121 ± 2	108 ± 4	110 ± 2	116 ± 4	113 ± 5
	2	124 ± 5	131 ± 4	113 ± 3	116 ± 5	130 ± 3	125 ± 3	116 ± 3	111 ± 2	126 ± 3	124 ± 5
	3	118 ± 4	128 ± 3	114 ± 2.	119 ± 3	119 ± 5	122 ± 3	114 ± 3	113 ± 2	122 ± 3	122 ± 4
	4	117 ± 4	128 ± 3	112 ± 3	107 ± 3	119 ± 3	123 ± 3	116 ± 3	114 ± 2	128 ± 3	113 ± 4

All values = mean ± SEM.
N = 16–18 for each strain/sex/cage/density group.
[a] For details of floor space for each density group, see Table 1.
All *P*-values compare Density 1 : Density 4: *$P < 0.05$; **$P < 0.005$; ***$P < 0.0005$
