# Supplementary material for: Effects of Housing Density in Five Inbred Strains of Mice
Source: PLoS One. 2014 Mar 21;9(3):e90012. doi: 10.1371/journal.pone.0090012 (PMC3962340; doi:10.1371/journal.pone.0090012)
Supplement: Table S4 — PercentFat,BMD131029. Percent fat (%) and areal bone mineral density (mg/cm2) for each of 5 strains for both the 3-month and 8-month timeframes. (PDF) [file pone.0090012.s006.pdf]

Table S4. Percent fat and areal bone mineral density.

Time-frame	Density group[a]	129S1/SvImJ		A/J		BALB/cByJ		C57BL/6J		DBA/2J	
		Duplex	Shoebox	Duplex	Shoebox	Duplex	Shoebox	Duplex	Shoebox	Duplex	Shoebox
**PERCENT FAT (%)**											
**Females**											
3-month	1	22.1 ± 0.4	25.8 ± 0.4	24.4 ± 0.6	24.6 ± 0.5	19.4 ± 0.3	19.0 ± 0.2	20.5 ± 0.3	20.9 ± 0.3	22.6 ± 0.4	23.5 ± 0.5
	2	23.9 ± 0.5	26.0 ± 0.5	24.7 ± 0.5	25.3 ± 0.6	19.6 ± 0.3	19.3 ± 0.2	21.2 ± 0.3	20.7 ± 0.4	24.1 ± 0.5	24.4 ± 0.6
	3	24.2 ± 0.6	24.8 ± 0.4	24.1 ± 0.4	24.8 ± 0.4	19.5 ± 0.4	19.7 ± 0.3	21.1 ± 0.3	21.9 ± 0.5	25.3 ± 0.6	25.6 ± 0.5
	4	24.2 ± 0.5*	24.9 ± 0.3	25.0 ± 0.5	25.0 ± 0.4	19.7 ± 0.2	19.5 ± 0.2	21.5 ± 0.4	21.8 ± 0.5	24.7 ± 0.3	24.6 ± 0.4
8-month	1	23.6 ± 0.4	23.9 ± 0.5	24.6 ± 0.9	27.3 ± 0.8	20.8 ± 0.4	22.9 ± 0.5	25.1 ± 1.0	26.3 ± 1.1	27.8 ± 1.2	32.5 ± 1.3
	2	24.5 ± 0.6	25.9 ± 0.6	28.6 ± 0.9	27.8 ± 1.0	20.3 ± 0.4	20.5 ± 0.6	22.9 ± 0.8	29.1 ± 1.3	28.7 ± 1.0	33.7 ± 1.2
	3	24.4 ± 0.6	26.8 ± 0.6	28.3 ± 0.8	28.9 ± 0.7	20.9 ± 0.4	20.4 ± 0.3	26.5 ± 1.0	26.3 ± 1.0	30.3 ± 1.1	34.9 ± 0.9
	4	25.6 ± 0.6	26.3 ± 0.6*	28.1 ± 0.6*	28.4 ± 1.0	20.7 ± 0.5	21.9 ± 0.5	25.3 ± 1.0	25.5 ± 0.9	30.5 ± 1.2	31.8 ± 0.8
**Males**											
3-month	1	21.4 ± 0.6	21.6 ± 0.4	22.2 ± 0.6	22.3 ± 0.5	17.6 ± 0.3	18.3 ± 0.4	19.0 ± 0.5	19.9 ± 0.6	24.5 ± 0.5	24.6 ± 0.6
	2	22.1 ± 0.3	23.1 ± 0.4	22.2 ± 0.5	22.3 ± 0.6	18.4 ± 0.4	17.6 ± 0.2	18.8 ± 0.6	19.6 ± 0.5	26.4 ± 0.6	26.2 ± 0.6
	3	22.0 ± 0.4	23.4 ± 0.5	24.4 ± 0.7	23.2 ± 0.4	18.0 ± 0.3	17.9 ± 0.3	19.1 ± 0.4	20.4 ± 0.7	26.6 ± 0.6	25.8 ± 0.5
	4	22.1 ± 0.5	22.7 ± 0.4	24.1 ± 0.7	22.7 ± 0.7	17.9 ± 0.3	17.7 ± 0.3	19.5 ± 0.5	20.6 ± 0.7	27.6 ± 0.6**	27.5 ± 0.5**
8-month	1	25.5 ± 1.1	24.0 ± 0.9	24.3 ± 0.9	26.1 ± 1.2	17.6 ± 0.6	19.4 ± 0.4	21.5 ± 0.9	24.2 ± 1.3	23.3 ± 1.0	19.4 ± 1.3
	2	24.7 ± 1.0	23.5 ± 0.8	23.7 ± 1.1	25.2 ± 1.1	18.2 ± 0.5	18.1 ± 0.6	24.1 ± 1.3	25.4 ± 1.1	19.5 ± 1.4	17.2 ± 0.9
	3	25.5 ± 1.3	27.7 ± 1.2	28.3 ± 1.1	25.8 ± 1.0	17.6 ± 0.6	17.6 ± 0.6	23.3 ± 1.3	26.1 ± 1.1	20.7 ± 1.3	17.7 ± 1.0
	4	25.3 ± 1.0	28.7 ± 0.9*	28.3 ± 1.3	24.6 ± 1.5	17.4 ± 0.5	18.4 ± 0.3	23.3 ± 1.0	25.8 ± 1.4	20.3 ± 1.3	18.8 ± 1.2
**AREAL BONE MINERAL DENSITY (mg/cm^2)**											
**Females**											
3-month	1	51.4 ± 0.5	49.2 ± 0.4	43.7 ± 0.4	44.4 ± 0.3	49.3 ± 0.3	49.1 ± 0.4	47.5 ± 0.2	47.8 ± 0.2	47.1 ± 0.2	47.0 ± 0.4
	2	52.3 ± 0.6	49.1 ± 0.8	43.5 ± 0.4	43.4 ± 0.2	49.6 ± 0.4	49.4 ± 0.3	47.0 ± 0.2	47.5 ± 0.3	46.5 ± 0.4	47.8 ± 0.4
	3	51.2 ± 0.6	50.8 ± 0.4	43.1 ± 0.3	43.1 ± 0.2	48.8 ± 0.4	49.7 ± 0.4	46.1 ± 0.3	46.6 ± 0.3	46.1 ± 0.4	47.7 ± 0.4
	4	51.6 ± 0.6	50.8 ± 0.6	43.2 ± 0.3	43.4 ± 0.4	49.3 ± 0.3	49.2 ± 0.4	46.9 ± 0.3	46.4 ± 0.3	46.8 ± 0.3	47.3 ± 0.3
8-month	1	57.6 ± 0.6	55.4 ± 0.7	46.2 ± 0.3	45.8 ± 0.3	54.0 ± 0.5	54.0 ± 0.4	49.8 ± 0.5	50.0 ± 0.3	49.3 ± 0.4	49.4 ± 0.4
	2	57.7 ± 0.6	55.6 ± 0.9	46.4 ± 0.3	45.4 ± 0.2	53.1 ± 0.5	53.9 ± 0.5	49.3 ± 0.3	51.2 ± 0.4	49.5 ± 0.4	49.9 ± 0.7
	3	57.3 ± 0.6	56.7 ± 0.8	45.8 ± 0.3	46.0 ± 0.4	53.2 ± 0.4	53.9 ± 0.4	48.6 ± 0.2	50.7 ± 0.3	49.5 ± 0.4	50.8 ± 0.4
	4	56.0 ± 0.6	56.2 ± 0.6	44.5 ± 0.2	44.9 ± 0.4	52.8 ± 0.3	54.6 ± 0.4	49.2 ± 0.4	50.4 ± 0.4	49.4 ± 0.3	51.0 ± 0.5
**Males**											
3-month	1	53.0 ± 0.7	50.5 ± 0.5	42.7 ± 0.3	42.4 ± 0.3	48.3 ± 0.3	48.9 ± 0.4	48.2 ± 0.3	48.7 ± 0.5	45.5 ± 0.3	46.1 ± 0.4
	2	51.9 ± 0.4	49.9 ± 0.5	43.2 ± 0.3	43.1 ± 0.3	48.6 ± 0.4	48.8 ± 0.2	48.2 ± 0.4	48.4 ± 0.5	45.3 ± 0.3	46.3 ± 0.5
	3	51.7 ± 0.5	51.0 ± 0.4	42.7 ± 0.2	42.6 ± 0.3	48.1 ± 0.2	48.2 ± 0.3	48.0 ± 0.4	48.0 ± 0.5	45.6 ± 0.4	46.8 ± 0.3
	4	52.9 ± 0.7	49.9 ± 0.5	42.3 ± 0.3	43.0 ± 0.3	48.4 ± 0.2	48.5 ± 0.3	47.8 ± 0.4	48.1 ± 0.5	46.0 ± 0.3	46.3 ± 0.5
8-month	1	58.1 ± 0.5	56.5 ± 0.9	46.2 ± 0.3	47.5 ± 0.3	52.9 ± 0.5	53.8 ± 0.3	51.0 ± 0.3	51.8 ± 0.4	50.2 ± 0.5	53.2 ± 0.6
	2	57.9 ± 0.8	57.9 ± 0.6	45.8 ± 0.2	46.0 ± 0.3	52.2 ± 0.4	53.7 ± 0.6	51.2 ± 0.3	52.2 ± 0.3	52.3 ± 0.4	53.7 ± 0.4
	3	58.0 ± 0.7	55.9 ± 0.5	45.7 ± 0.5	46.0 ± 0.4	52.5 ± 0.4	53.5 ± 0.4	51.1 ± 0.3	52.4 ± 0.4	51.5 ± 0.5	55.2 ± 0.4
	4	56.0 ± 0.6	55.5 ± 0.5	45.7 ± 0.3	45.7 ± 0.2	51.6 ± 0.6	53.5 ± 0.3	50.8 ± 0.4	51.3 ± 0.5	51.6 ± 0.5	53.1 ± 0.5

All values = mean ± SEM.
N = 16–18 for each strain/sex/cage/density group.
[a] For details of floor space for each density group, see Table 1.
All *P*-values compare Density 1 : Density 4: *$P < 0.05$; **$P < 0.005$
