# Supplementary material for: Effects of Housing Density in Five Inbred Strains of Mice
Source: PLoS One. 2014 Mar 21;9(3):e90012. doi: 10.1371/journal.pone.0090012 (PMC3962340; doi:10.1371/journal.pone.0090012)
Supplement: Table S5 — Cholesterol131029. HDL and total cholesterol (mg/dL) for each of 5 strains for both the 3-month and 8-month timeframes. (PDF) [file pone.0090012.s007.pdf]

Table S5. HDL and total cholesterol (mg/dL).

Density group	129S1/SvImJ				A/J				BALB/cByJ			
	HDL		Total		HDL		Total		HDL		Total	
	Duplex	Shoebox	Duplex	Shoebox	Duplex	Shoebox	Duplex	Shoebox	Duplex	Shoebox	Duplex	Shoebox
**FEMALES**												
3-month												
1	65 ± 2	72 ± 3	93 ± 2	93 ± 3	38 ± 2	46 ± 2	65 ± 2	64 ± 2	43 ± 3	54 ± 3	64 ± 3	70 ± 3
2	67 ± 2	75 ± 2	97 ± 1	99 ± 2	37 ± 1	46 ± 2	58 ± 2	65 ± 2	40 ± 3	55 ± 1	59 ± 3	72 ± 3
3	66 ± 2	74 ± 4	96 ± 2	100 ± 4	38 ± 2	43 ± 2	64 ± 2	64 ± 2	41 ± 2	52 ± 2	61 ± 2	69 ± 3
4	63 ± 1	72 ± 3	96 ± 2	99 ± 3	39 ± 2	43 ± 2	61 ± 1	63 ± 1	45 ± 1	56 ± 2	63 ± 2	69 ± 3
8-month												
1	74 ± 2	70 ± 2	95 ± 3	97 ± 3	45 ± 1	46 ± 1	58 ± 1	61 ± 1	46 ± 2	47 ± 3	61 ± 1	57 ± 2
2	76 ± 3	72 ± 2	93 ± 3	97 ± 2	45 ± 1	47 ± 2	58 ± 2	61 ± 2	41 ± 2	48 ± 3	56 ± 2	59 ± 3
3	77 ± 4	70 ± 2	98 ± 4	89 ± 2	43 ± 2	46 ± 2	60 ± 1	64 ± 2	46 ± 2	49 ± 2	62 ± 2	58 ± 2
4	71 ± 2	75 ± 2	93 ± 3	96 ± 3	42 ± 1	47 ± 1	57 ± 1	60 ± 2	46 ± 1	51 ± 2	59 ± 2	59 ± 2
**MALES**												
3-month												
1	89 ± 3	95 ± 2	114 ± 3	115 ± 2	45 ± 1	47 ± 2	65 ± 1	64 ± 1	64 ± 4	78 ± 3	80 ± 3	88 ± 3
2	89 ± 3	95 ± 3	114 ± 3	120 ± 3	46 ± 2	45 ± 1	66 ± 1	64 ± 1	70 ± 2	79 ± 4	82 ± 2	91 ± 3
3	90 ± 4	96 ± 3	112 ± 3	119 ± 3	46 ± 2	45 ± 2	69 ± 2	64 ± 1	67 ± 2	76 ± 4	83 ± 2	88 ± 3
4	93 ± 3	93 ± 2	117 ± 3	118 ± 3	47 ± 1	42 ± 1	66 ± 1	60 ± 2	77 ± 2	73 ± 3	85 ± 2	85 ± 3
8-month												
1	99 ± 4	92 ± 2	116 ± 4	122 ± 4	51 ± 1	51 ± 2	65 ± 2	66 ± 1	77 ± 3	77 ± 3	91 ± 2	84 ± 3
2	97 ± 3	93 ± 3	115 ± 3	113 ± 3	53 ± 1	54 ± 1	63 ± 2	66 ± 2	81 ± 2	77 ± 2	92 ± 3	85 ± 2
3	94 ± 5	102 ± 3	120 ± 3	125 ± 3	53 ± 2	54 ± 2	66 ± 2	71 ± 2	78 ± 4	76 ± 4	84 ± 4	86 ± 5
4	91 ± 4	107 ± 3	112 ± 4	126 ± 4	52 ± 2	55 ± 2	67 ± 3	69 ± 3	79 ± 4	85 ± 3	91 ± 4	88 ± 3

Density group	C57BL/6J				DBA/2J			
	HDL		Total		HDL		Total	
	Duplex	Shoebox	Duplex	Shoebox	Duplex	Shoebox	Duplex	Shoebox
**FEMALES**								
3-month								
1	41 ± 2	47 ± 1	67 ± 2	66 ± 2	41 ± 2	46 ± 1	71 ± 2	74 ± 1
2	41 ± 2	45 ± 2	61 ± 2	67 ± 2	40 ± 3	47 ± 2	69 ± 3	76 ± 2
3	38 ± 2	47 ± 1	65 ± 2	68 ± 2	40 ± 3	46 ± 2	73 ± 3	77 ± 3
4	41 ± 1	44 ± 2	62 ± 1	66 ± 2	40 ± 3	42 ± 1	71 ± 2	73 ± 1
8-month								
1	39 ± 2	46 ± 3	62 ± 2	60 ± 3	37 ± 2	40 ± 3	55 ± 3	63 ± 3
2	40 ± 2	48 ± 2	63 ± 2	62 ± 2	33 ± 2	42 ± 2	56 ± 3	60 ± 2
3	37 ± 2	46 ± 1	62 ± 1	63 ± 2	33 ± 3	41 ± 1	51 ± 2	68 ± 3
4	40 ± 1	46 ± 2	63 ± 2	59 ± 2	37 ± 3	46 ± 2	59 ± 2	66 ± 3
**MALES**								
3-month								
1	57 ± 2	67 ± 2	83 ± 2	85 ± 3	57 ± 2	69 ± 2	89 ± 2	94 ± 3
2	59 ± 2	67 ± 1	84 ± 2	88 ± 2	65 ± 2	71 ± 2	94 ± 2	102 ± 3
3	61 ± 1	73 ± 2	85 ± 2	94 ± 2	63 ± 2	70 ± 2	94 ± 2	96 ± 3
4	61 ± 2	72 ± 2	85 ± 1	93 ± 2	65 ± 3	74 ± 2	97 ± 3	104 ± 2
8-month								
1	58 ± 2	68 ± 3	83 ± 2	84 ± 2	66 ± 2	71 ± 2	90 ± 2	101 ± 2
2	61 ± 3	70 ± 2	83 ± 2	84 ± 2	66 ± 2	70 ± 2	88 ± 2	96 ± 3
3	62 ± 2	69 ± 3	85 ± 2	83 ± 3	66 ± 1	67 ± 3	93 ± 3	97 ± 4
4	61 ± 2	68 ± 3	82 ± 2	86 ± 3	70 ± 3	73 ± 3	99 ± 3	97 ± 3

All values = mean ± SEM.
N = 16–18 for each strain/sex/cage/density group.
[a] For details of floor space for each density group, see Table 1.
