# Supplementary material for: Effects of Housing Density in Five Inbred Strains of Mice
Source: PLoS One. 2014 Mar 21;9(3):e90012. doi: 10.1371/journal.pone.0090012 (PMC3962340; doi:10.1371/journal.pone.0090012)
Supplement: Table S6 — Heart, TestesWt131029. Heart weight and testes weight (mg) for each of 5 strains for both the 3-month and 8-month timeframes. (PDF) [file pone.0090012.s008.pdf]

Table S6. Heart weight and testes weight.

Time-frame	Density group[a]	129S1/SvImJ Duplex	129S1/SvImJ Shoebox	A/J Duplex	A/J Shoebox	BALB/cByJ Duplex	BALB/cByJ Shoebox	C57BL/6J Duplex	C57BL/6J Shoebox	DBA/2J Duplex	DBA/2J Shoebox
**HEART WEIGHT (mg)**											
**Females**											
3-month	1	89 ± 1	78 ± 2	91 ± 2	92 ± 2	104 ± 2	103 ± 2	99 ± 3	92 ± 2	115 ± 3	105 ± 3
	2	86 ± 2	81 ± 2	91 ± 2	87 ± 2	102 ± 2	113 ± 3	96 ± 2	93 ± 2	119 ± 3	114 ± 2
	3	83 ± 2	87 ± 2	94 ± 4	86 ± 2	105 ± 2	110 ± 2	96 ± 2	91 ± 2	118 ± 3	113 ± 3
	4	86 ± 2	84 ± 2	92 ± 3	86 ± 1	106 ± 2	108 ± 2	94 ± 3	90 ± 1	118 ± 4	110 ± 2
8-month	1	98 ± 3	92 ± 2	97 ± 3	96 ± 2	115 ± 2	113 ± 2	108 ± 3	104 ± 2	117 ± 3	127 ± 4
	2	97 ± 2	91 ± 3	93 ± 2	92 ± 2	109 ± 2	114 ± 2	104 ± 2	107 ± 2	121 ± 4	126 ± 4
	3	96 ± 3	94 ± 3	91 ± 2	95 ± 2	109 ± 2	114 ± 3	101 ± 3	112 ± 2	126 ± 3	131 ± 3
	4	96 ± 3	88 ± 2	91 ± 2	92 ± 2	108 ± 1	116 ± 2	100 ± 2	105 ± 2	122 ± 3	128 ± 4
**Males**											
3-month	1	126 ± 5	112 ± 3	102 ± 2	96 ± 2	138 ± 4	142 ± 2	146 ± 6	131 ± 3	144 ± 3.	137 ± 4
	2	114 ± 3	108 ± 3	106 ± 3	96 ± 2	140 ± 4	142 ± 1	144 ± 6	125 ± 4	144 ± 4	146 ± 4
	3	121 ± 4	114 ± 3	97 ± 2	96 ± 2	139 ± 3	139 ± 2	147 ± 6	123 ± 3	150 ± 4	137 ± 4
	4	122 ± 4	106 ± 3	97 ± 2	101 ± 2	143 ± 5	134 ± 2	138 ± 5	122 ± 3	145 ± 4	139 ± 3
8-month	1	139 ± 3	129 ± 3	107 ± 3	106 ± 3	163 ± 3	163 ± 2	148 ± 4	144 ± 4	179 ± 6	179 ± 3
	2	139 ± 5	135 ± 4	107 ± 3	104 ± 3	164 ± 3	164 ± 3	143 ± 3	153 ± 3	179 ± 5	184 ± 4
	3	135 ± 3	124 ± 5	108 ± 4	110 ± 3	162 ± 2	155 ± 4	139 ± 4	150 ± 2	190 ± 7.	193 ± 5
	4	131 ± 4	129 ± 4	107 ± 3	117 ± 3	166 ± 4	158 ± 3	139 ± 2	139 ± 4	190 ± 7	189 ± 5
**TESTES WEIGHT (mg)**											
**Males**											
3-month	1	205 ± 4	194 ± 5	143 ± 3	152 ± 3	198 ± 3	189 ± 6	200 ± 3	196 ± 3	211 ± 3	216 ± 4
	2	202 ± 4	187 ± 7	148 ± 2	144 ± 2	194 ± 3	204 ± 4	205 ± 4	194 ± 3	212 ± 3	218 ± 4
	3	213 ± 5	188 ± 6	147 ± 3	144 ± 3	193 ± 3	194 ± 3	204 ± 4	194 ± 3	210 ± 4	207 ± 5
	4	210 ± 6	189 ± 6	150 ± 3	147 ± 4	202 ± 4	194 ± 3	199 ± 3	194 ± 4	208 ± 3	215 ± 4
8-month	1	224 ± 6	217 ± 5	147 ± 3	151 ± 3	207 ± 5	207 ± 5	202 ± 3	200 ± 4	215 ± 6	219 ± 6
	2	208 ± 8	206 ± 7	146 ± 3	148 ± 3	202 ± 4	204 ± 6	205 ± 4	201 ± 4	205 ± 6	208 ± 7
	3	206 ± 8	219 ± 5	151 ± 3	150 ± 3	205 ± 3	195 ± 5	211 ± 4	209 ± 4	200 ±10	176 ± 9
	4	212 ± 4	221 ± 6	156 ± 4	151 ± 3	208 ± 5	209 ± 6	210 ± 3	203 ± 3	204 ± 8	164 ± 5

All values = mean ± SEM.
N = 16–18 for each strain/sex/cage/density group.
[a] For details of floor space for each density group, see Table 1.
