# Supplementary material for: Effects of Housing Density in Five Inbred Strains of Mice
Source: PLoS One. 2014 Mar 21;9(3):e90012. doi: 10.1371/journal.pone.0090012 (PMC3962340; doi:10.1371/journal.pone.0090012)
Supplement: Table S7 — RBC,WBC121029. Red (×106/µL) and while blood cell counts (×103/µL) for each of 5 strains for both the 3-month and 8-month timeframes. (PDF) [file pone.0090012.s009.pdf]

Table S7. Red and white blood cell counts.

Time-frame	Density group[a]	129S1/SvImJ		A/J		BALB/cByJ		C57BL/6J		DBA/2J	
		Duplex	Shoebox	Duplex	Shoebox	Duplex	Shoebox	Duplex	Shoebox	Duplex	Shoebox
**RED BLOOD CELLS (x10^6/µL)**											
**Females**											
3-month	1	11.0 ± 0.1	11.0 ± 0.1	10.2 ± 0.1	10.0 ± 0.1	10.7 ± 0.1	10.4 ± 0.1	10.6 ± 0.1	10.4 ± 0.1	11.2 ± 0.1	11.1 ± 0.1
	2	11.0 ± 0.1	10.9 ± 0.1	10.2 ± 0.1	10.0 ± 0.1	10.8 ± 0.1	10.5 ± 0.1	10.7 ± 0.1	10.6 ± 0.1	11.3 ± 0.1	11.2 ± 0.1
	3	10.9 ± 0.1	10.9 ± 0.1	10.1 ± 0.1	10.1 ± 0.1	10.6 ± 0.1	10.4 ± 0.1	10.7 ± 0.1	10.5 ± 0.1	11.2 ± 0.1	11.2 ± 0.1
	4	11.0 ± 0.1	10.8 ± 0.1	10.1 ± 0.1	10.1 ± 0.1	10.8 ± 0.1	10.3 ± 0.1	10.7 ± 0.1	10.4 ± 0.1	11.3 ± 0.1	11.1 ± 0.1
8-month	1	11.1 ± 0.1	11.4 ± 0.1	9.8 ± 0.1	9.7 ± 0.1	10.9 ± 0.1	10.6 ± 0.1	10.9 ± 0.1	10.5 ± 0.1	11.1 ± 0.1	11.1 ± 0.1
	2	11.1 ± 0.1	11.3 ± 0.1	9.8 ± 0.1	9.8 ± 0.1	11.0 ± 0.1	10.6 ± 0.1	10.9 ± 0.1	10.5 ± 0.1	11.2 ± 0.1	11.0 ± 0.1
	3	11.1 ± 0.1	11.2 ± 0.1	9.8 ± 0.1	9.8 ± 0.1	10.9 ± 0.1	10.5 ± 0.1	10.9 ± 0.1	10.5 ± 0.1	11.1 ± 0.1	10.9 ± 0.1
	4	11.1 ± 0.1	11.2 ± 0.1	9.9 ± 0.1	9.7 ± 0.1	10.9 ± 0.1	10.5 ± 0.1	10.8 ± 0.1	10.4 ± 0.1	11.1 ± 0.1	11.0 ± 0.1
Males											
3-month	1	10.9 ± 0.1	10.7 ± 0.1	10.3 ± 0.1	10.1 ± 0.1	10.7 ± 0.1	10.7 ± 0.1	10.8 ± 0.1	10.6 ± 0.1	11.2 ± 0.1	11.3 ± 0.1
	2	11.0 ± 0.1	11.1 ± 0.1	10.0 ± 0.1	10.1 ± 0.1	10.9 ± 0.1	10.8 ± 0.1	10.7 ± 0.1	10.6 ± 0.1	11.2 ± 0.1	11.2 ± 0.1
	3	10.9 ± 0.1	10.8 ± 0.1	10.2 ± 0.1	10.1 ± 0.1	10.7 ± 0.1	10.7 ± 0.1	10.6 ± 0.1	10.5 ± 0.1	11.1 ± 0.1	11.3 ± 0.1
	4	11.0 ± 0.1	10.9 ± 0.1	10.3 ± 0.1	10.2 ± 0.1	10.9 ± 0.1	10.5 ± 0.1	10.6 ± 0.1	10.5 ± 0.1	11.4 ± 0.1	11.3 ± 0.1
8-month	1	11.2 ± 0.1	11.3 ± 0.1	9.9 ± 0.1	10.1 ± 0.1	11.1 ± 0.1	10.7 ± 0.1	10.7 ± 0.1	10.3 ± 0.1	11.1 ± 0.1	11.0 ± 0.1
	2	11.1 ± 0.1	11.2 ± 0.1	9.9 ± 0.1	10.1 ± 0.1	11.0 ± 0.1	10.5 ± 0.1	10.7 ± 0.1	10.3 ± 0.1	11.7 ± 0.1	11.0 ± 0.1
	3	11.2 ± 0.1	11.4 ± 0.1	9.8 ± 0.1	10.1 ± 0.1	11.0 ± 0.1	10.5 ± 0.1	10.7 ± 0.1	10.2 ± 0.1	11.1 ± 0.1	11.0 ± 0.1
	4	11.1 ± 0.1	11.3 ± 0.1	9.9 ± 0.1	9.9 ± 0.1	10.9 ± 0.1	10.5 ± 0.1	10.5 ± 0.1	10.2 ± 0.1	11.6 ± 0.1	11.0 ± 0.1
**WHITE BLOOD CELLS (x10^3/µL)**											
**Females**											
3-month	1	11.2 ± 0.6	11.0 ± 0.5	6.8 ± 0.3	6.7 ± 0.4	8.6 ± 0.3	8.6 ± 0.4	9.4 ± 0.4	8.5 ± 0.5	10.7 ± 0.7	11.0 ± 0.7
	2	11.1 ± 0.4	10.7 ± 0.6	7.7 ± 0.3	5.9 ± 0.4	8.1 ± 0.4	8.3 ± 0.3	8.3 ± 0.3	8.6 ± 0.4	10.0 ± 0.6	10.3 ± 0.7
	3	12.1 ± 0.4	11.1 ± 0.6	6.5 ± 0.3	6.6 ± 0.3	8.9 ± 0.3	8.1 ± 0.3	9.8 ± 0.4	8.6 ± 0.5	10.1 ± 0.6	10.6 ± 0.7
	4	11.9 ± 0.5	11.6 ± 0.5	7.4 ± 0.4	6.4 ± 0.4	8.2 ± 0.3	8.4 ± 0.3	9.3 ± 0.3	8.6 ± 0.4	9.8 ± 0.4	11.0 ± 0.5
8-month	1	9.6 ± 0.5	11.6 ± 0.5	5.7 ± 0.3	5.8 ± 0.2	6.3 ± 0.2	6.9 ± 0.3	7.5 ± 0.5	8.8 ± 0.4	8.0 ± 0.4	9.7 ± 0.5
	2	10.3 ± 0.6	10.5 ± 0.6	6.4 ± 0.3	5.4 ± 0.2	6.7 ± 0.2	6.9 ± 0.4	7.1 ± 0.5	9.5 ± 0.5	8.7 ± 0.6	11.1 ± 0.5
	3	10.3 ± 0.5	9.9 ± 0.5	6.2 ± 0.3	6.0 ± 0.3	6.4 ± 0.3	5.9 ± 0.3	8.2 ± 0.4	8.6 ± 0.3	8.3 ± 0.5	10.9 ± 0.5
	4	10.0 ± 0.6	9.8 ± 0.4	6.7 ± 0.5	6.7 ± 0.3	6.5 ± 0.2	6.3 ± 0.3	7.8 ± 0.4	9.6 ± 0.5	8.0 ± 0.5	11.2 ± 0.5
**Males**											
3-month	1	10.4 ± 0.3	10.6 ± 0.5	7.4 ± 0.4	7.2 ± 0.4	9.5 ± 0.3	8.6 ± 0.3	9.9 ± 0.4	9.4 ± 0.4	13.2 ± 0.5	11.8 ± 0.5
	2	10.6 ± 0.5	11.3 ± 0.5	7.7 ± 0.4	7.1 ± 0.4	8.7 ± 0.4	8.1 ± 0.3	9.0 ± 0.4	9.4 ± 0.4	12.7 ± 0.5	10.6 ± 0.4
	3	9.1 ± 0.3	11.2 ± 0.5	7.3 ± 0.3	6.7 ± 0.5	8.8 ± 0.3	8.5 ± 0.4	9.5 ± 0.3	9.0 ± 0.5	13.0 ± 0.8	10.9 ± 0.5
	4	10.1 ± 0.5	10.9 ± 0.4	7.3 ± 0.5	7.3 ± 0.4	8.6 ± 0.3	7.7 ± 0.3	10.0 ± 0.5	9.4 ± 0.4	13.9 ± 0.8	11.1 ± 0.4
8-month	1	7.4 ± 0.3	9.5 ± 0.4	6.3 ± 0.3	7.8 ± 0.4	6.9 ± 0.3	7.1 ± 0.3	8.5 ± 0.7	10.9 ± 0.7	6.7 ± 0.6	9.7 ± 0.5
	2	8.0 ± 0.5	9.1 ± 0.4	6.6 ± 0.3	7.3 ± 0.3	7.6 ± 0.5	6.9 ± 0.2	8.9 ± 0.5	9.7 ± 0.8	8.1 ± 0.8	10.5 ± 0.6
	3	8.7 ± 0.5	9.0 ± 0.3	6.0 ± 0.3	8.1 ± 0.5	6.5 ± 0.4	6.8 ± 0.5	7.8 ± 0.6	10.6 ± 0.5	9.3 ± 0.7	11.1 ± 0.5
	4	9.6 ± 0.7	9.5 ± 0.3	6.4 ± 0.3	7.9 ± 0.3	6.9 ± 0.3	6.6 ± 0.4	8.4 ± 0.6	10.6 ± 0.5	8.5 ± 0.6	12.8 ± 0.5

All values = mean ± SEM.
N = 16–18 for each strain/sex/cage/density group.
[a] For details of floor space for each density group, see Table 1.
