# Supplementary material for: Effects of Housing Density in Five Inbred Strains of Mice
Source: PLoS One. 2014 Mar 21;9(3):e90012. doi: 10.1371/journal.pone.0090012 (PMC3962340; doi:10.1371/journal.pone.0090012)
Supplement: Table S8 — Hemoglobin, Heatrocrit131029. Hemoglobin (g/dL) and hematocrit (% red blood cells/total volume of blood) for each of 5 strains for both the 3-month and 8-month timeframes. (PDF) [file pone.0090012.s010.pdf]

Table S8. Hemoglobin and hematocrit.

Time-frame	Density group[a]	129S1/SvImJ Duplex	129S1/SvImJ Shoebox	A/J Duplex	A/J Shoebox	BALB/cByJ Duplex	BALB/cByJ Shoebox	C57BL/6J Duplex	C57BL/6J Shoebox	DBA/2J Duplex	DBA/2J Shoebox
**HEMOGLOBIN (g/dL)**											
**Females**											
3-month	1	17.5 ± 0.1	17.7 ± 0.1	15.1 ± 0.1	15.0 ± 0.1	16.8 ± 0.1	16.6 ± 0.1	16.4 ± 0.1	16.1 ± 0.1	14.9 ± 0.1	15.1 ± 0.1
	2	17.6 ± 0.1	17.6 ± 0.1	15.2 ± 0.1	15.1 ± 0.1	16.9 ± 0.1	16.6 ± 0.1	16.5 ± 0.1	16.5 ± 0.1	15.0 ± 0.1	15.3 ± 0.1
	3	17.4 ± 0.1	17.7 ± 0.1	14.9 ± 0.1	15.3 ± 0.1	16.7 ± 0.1	16.5 ± 0.1	16.4 ± 0.1	16.1 ± 0.1	14.9 ± 0.1	15.2 ± 0.1
	4	17.4 ± 0.1	17.5 ± 0.1	15.0 ± 0.1	15.2 ± 0.1	16.9 ± 0.1	16.5 ± 0.1	16.5 ± 0.1	16.2 ± 0.1	15.1 ± 0.1	15.1 ± 0.1
8-month	1	17.2 ± 0.1	17.3 ± 0.1	14.9 ± 0.1	14.9 ± 0.1	16.6 ± 0.1	16.3 ± 0.1	16.0 ± 0.1	15.8 ± 0.1	15.0 ± 0.1	15.1 ± 0.1
	2	17.1 ± 0.1	17.3 ± 0.1	15.0 ± 0.1	14.8 ± 0.1	16.6 ± 0.1	16.4 ± 0.1	16.1 ± 0.1	15.9 ± 0.1	15.2 ± 0.1	15.1 ± 0.1
	3	17.2 ± 0.1	17.2 ± 0.1	15.1 ± 0.1	14.9 ± 0.1	16.5 ± 0.1	16.2 ± 0.1	15.9 ± 0.1	15.7 ± 0.1	15.2 ± 0.1	15.1 ± 0.1
	4	17.1 ± 0.1	17.2 ± 0.1	14.9 ± 0.1	14.7 ± 0.1	16.4 ± 0.1	16.2 ± 0.1	15.9 ± 0.1	15.7 ± 0.1	15.2 ± 0.1	15.3 ± 0.1
Males											
3-month	1	17.4 ± 0.1	17.3 ± 0.1	15.1 ± 0.1	15.1 ± 0.1	16.7 ± 0.1	16.9 ± 0.1	16.2 ± 0.1	16.4 ± 0.1	15.0 ± 0.1	15.4 ± 0.1
	2	17.4 ± 0.1	17.8 ± 0.1	14.8 ± 0.1	15.0 ± 0.1	16.9 ± 0.0	17.0 ± 0.1	16.0 ± 0.1	16.2 ± 0.1	14.7 ± 0.2	15.2 ± 0.1
	3	17.4 ± 0.1	17.5 ± 0.1	14.9 ± 0.1	15.0 ± 0.1	16.6 ± 0.1	16.9 ± 0.1	16.0 ± 0.1	16.2 ± 0.1	14.9 ± 0.1	15.5 ± 0.2
	4	17.5 ± 0.1	17.7 ± 0.1	15.1 ± 0.1	15.2 ± 0.1	16.8 ± 0.1	16.7 ± 0.1	16.0 ± 0.1	16.1 ± 0.1	15.1 ± 0.1	15.3 ± 0.2
8-month	1	17.0 ± 0.1	17.2 ± 0.1	14.5 ± 0.1	14.7 ± 0.1	16.5 ± 0.1	16.2 ± 0.1	15.5 ± 0.1	15.2 ± 0.1	14.8 ± 0.2	15.0 ± 0.2
	2	17.0 ± 0.1	17.1 ± 0.1	14.6 ± 0.1	14.6 ± 0.1	16.6 ± 0.1	15.9 ± 0.1	15.5 ± 0.1	15.4 ± 0.1	15.2 ± 0.1	15.4 ± 0.2
	3	17.0 ± 0.1	17.2 ± 0.1	14.5 ± 0.1	14.8 ± 0.1	16.3 ± 0.1	16.0 ± 0.1	15.5 ± 0.1	15.2 ± 0.1	15.0 ± 0.2	14.9 ± 0.1
	4	16.9 ± 0.1	17.2 ± 0.1	14.6 ± 0.1	14.8 ± 0.1	16.2 ± 0.1	15.9 ± 0.1	15.3 ± 0.1	15.3 ± 0.1	15.3 ± 0.1	15.0 ± 0.2
**HEMATOCRIT (% red blood cells / total volume of blood)**											
**Females**											
3-month	1	53.7 ± 0.3	53.9 ± 0.4	46.2 ± 0.3	46.1 ± 0.3	50.2 ± 0.4	49.9 ± 0.4	51.4 ± 0.3	51.4 ± 0.4	47.6 ± 0.5	47.9 ± 0.3
	2	53.3 ± 0.2	53.3 ± 0.5	46.2 ± 0.4	45.9 ± 0.4	50.0 ± 0.3	49.9 ± 0.3	51.4 ± 0.4	52.4 ± 0.3	47.6 ± 0.3	48.3 ± 0.5
	3	52.9 ± 0.4	53.7 ± 0.5	45.9 ± 0.3	46.8 ± 0.3	49.5 ± 0.4	49.6 ± 0.3	51.6 ± 0.3	51.5 ± 0.3	47.2 ± 0.4	48.0 ± 0.3
	4	53.0 ± 0.4	53.2 ± 0.4	45.8 ± 0.4	46.5 ± 0.4	49.9 ± 0.4	49.5 ± 0.3	51.6 ± 0.3	51.3 ± 0.4	48.0 ± 0.4	47.7 ± 0.5
8-month	1	51.7 ± 0.3	51.7 ± 0.3	44.3 ± 0.3	42.9 ± 0.4	49.5 ± 0.3	47.3 ± 0.2	50.9 ± 0.3	48.1 ± 0.3	47.1 ± 0.5	44.8 ± 0.4
	2	51.4 ± 0.3	51.4 ± 0.3	44.4 ± 0.4	43.6 ± 0.5	49.8 ± 0.3	47.5 ± 0.4	51.1 ± 0.3	48.5 ± 0.3	46.7 ± 0.4	44.9 ± 0.5
	3	51.5 ± 0.4	51.0 ± 0.3	44.3 ± 0.3	43.5 ± 0.5	49.1 ± 0.3	47.0 ± 0.2	50.9 ± 0.3	47.8 ± 0.4	46.5 ± 0.4	45.3 ± 0.3
	4	51.5 ± 0.3	50.8 ± 0.2	44.0 ± 0.4	43.4 ± 0.4	49.0 ± 0.2	46.8 ± 0.3	50.5 ± 0.3	47.9 ± 0.3	47.2 ± 0.4	45.3 ± 0.6
**Males**											
3-month	1	53.0 ± 0.4	53.0 ± 0.4	47.0 ± 0.4	46.6 ± 0.3	50.4 ± 0.4	51.5 ± 0.4	53.0 ± 0.3	53.4 ± 0.4	48.4 ± 0.5	49.3 ± 0.6
	2	52.9 ± 0.5	54.7 ± 0.4	45.5 ± 0.4	46.0 ± 0.4	50.7 ± 0.3	51.7 ± 0.3	52.1 ± 0.4	52.2 ± 0.3	48.1 ± 0.5	49.1 ± 0.5
	3	52.7 ± 0.5	53.7 ± 0.4	46.3 ± 0.5	46.3 ± 0.2	50.2 ± 0.5	50.9 ± 0.5	52.4 ± 0.3	52.5 ± 0.3	47.5 ± 0.5	49.8 ± 0.5
	4	53.0 ± 0.4	54.0 ± 0.4	46.9 ± 0.3	46.6 ± 0.3	50.5 ± 0.3	50.9 ± 0.4	51.9 ± 0.2	52.2 ± 0.3	48.6 ± 0.5	49.1 ± 0.6
8-month	1	51.2 ± 0.3	50.7 ± 0.4	43.7 ± 0.4	43.7 ± 0.4	50.0 ± 0.2	47.9 ± 0.3	50.7 ± 0.5	47.5 ± 0.3	46.5 ± 0.7	45.5 ± 0.6
	2	50.8 ± 0.3	50.2 ± 0.4	44.0 ± 0.4	44.0 ± 0.4	50.4 ± 0.2	46.9 ± 0.3	50.1 ± 0.4	47.8 ± 0.4	48.5 ± 0.8	45.9 ± 0.6
	3	50.9 ± 0.3	50.8 ± 0.3	43.4 ± 0.3	44.1 ± 0.3	49.7 ± 0.3	47.0 ± 0.2	50.9 ± 0.6	47.4 ± 0.3	46.5 ± 0.5	45.0 ± 0.7
	4	50.6 ± 0.2	51.0 ± 0.4	43.7 ± 0.3	43.8 ± 0.4	49.2 ± 0.4	46.8 ± 0.3	49.7 ± 0.6	47.5 ± 0.4	48.3 ± 0.5	45.6 ± 0.6

All values = mean ± SEM.
N = 16–18 for each strain/sex/cage/density group.
[a] For details of floor space for each density group, see Table 1.
