# Supplementary material for: Effects of Housing Density in Five Inbred Strains of Mice
Source: PLoS One. 2014 Mar 21;9(3):e90012. doi: 10.1371/journal.pone.0090012 (PMC3962340; doi:10.1371/journal.pone.0090012)
Supplement: Table S9 — Platelets,Lymphocytes131029. Platelet count (×103/µL) and lymphocyte count (% white blood cells) for each of 5 strains for both the 3-month and 8-month timeframes. (PDF) [file pone.0090012.s011.pdf]

Table S9. Platelet count and lymphocyte count.

Time-frame	Density group[a]	129S1/SvImJ		A/J		BALB/cByJ		C57BL/6J		DBA/2J	
		Duplex	Shoebox	Duplex	Shoebox	Duplex	Shoebox	Duplex	Shoebox	Duplex	Shoebox
**PLATELET COUNT (x10^3/µL)**											
**Females**											
3-month	1	759 ± 22	784± 25	1024 ± 28	1030 ± 31	1095 ± 26	1135 ± 22	1105 ± 25	1201 ± 20	1043 ± 42	1052 ± 68
	2	742 ± 27	826 ± 39	1014 ± 29	985 ± 28	1132 ± 30	1148 ± 35	1100 ± 28	1164 ± 25	1043 ± 42	902 ± 74
	3	807 ± 25	810 ± 28	1036 ± 30	1059 ± 27	1194 ± 27	1139 ± 29	1075 ± 36	1175 ± 28	926 ± 47	1059 ± 65
	4	821 ± 20	885 ± 29	1081 ± 26	1004 ± 25	1183 ± 31	1195 ± 27	1090 ± 30	1200 ± 28	978 ± 77	878 ± 75
8-month	1	942 ± 25	946 ± 29	1129 ± 30	1157 ± 41	1102 ± 40	1072 ± 25	1165 ± 36	1097 ± 34	1037 ± 34	1055 ± 28
	2	984 ± 18	1018 ± 28	1200 ± 36	1170 ± 29	1144 ± 31	1105 ± 21	1184 ± 35	1086 ± 28	1030 ± 39	1032 ± 39
	3	955 ± 23	977 ± 33	1163 ± 48	1185 ± 33	1189 ± 31	1127 ± 19	1148 ± 30	1157 ± 25	1077 ± 45	1055 ± 25
	4	979 ± 21	1018 ± 23	1188 ± 32	1148 ± 36	1163 ± 32	1122 ± 21	1199 ± 32	1132 ± 19	1092 ± 31	1052 ± 41
Males											
3-month	1	955 ± 14	948 ± 34	1087 ± 48	1078 ± 25	1262 ± 21	1326 ± 23	1245 ± 31	1322 ± 30	1039 ± 52	1048 ± 54
	2	923 ± 31	900 ± 21	1175 ± 38	1060 ± 32	1235 ± 27	1306 ± 15	1263 ± 30	1381 ± 31	1174 ± 46	1027 ± 69
	3	973 ± 21	973 ± 33	1075 ± 41	1116 ± 34	1201 ± 32	1285 ± 27	1184 ± 19	1356 ± 36	1134 ± 41	999 ± 56
	4	972 ± 23	958 ± 26	1132 ± 32	1153 ± 35	1220 ± 34	1311 ± 30	1257 ± 39	1348 ± 35	1121 ± 47	1100 ± 59
8-month	1	1041 ± 23	1114 ± 24	1157 ± 38	1095 ± 41	1294 ± 29	1210 ± 32	1353 ± 30	1354 ± 34	980 ± 49	947 ± 35
	2	1048 ± 24	1077 ± 20	1173 ± 34	1030 ± 41	1310 ± 23	1194 ± 36	1405 ± 24	1348 ± 34	955 ± 61	1009 ± 35
	3	1053 ± 24	1133 ± 22	1208 ± 35	1065 ± 29	1345 ± 40	1218 ± 40	1428 ± 28	1404 ± 22	1046 ± 32	1064 ± 53
	4	1051 ± 36	1075 ± 20	1200 ± 52	1106 ± 29	1304 ± 34	1222 ± 24	1371 ± 21	1358 ± 30	1026 ± 42	1019 ± 40
**LYMPHOCYTE COUNT (% white blood cells)**											
**Females**											
3-month	1	82.2 ± 0.7	84.3 ± 0.6	79.6 ± 0.6	79.7 ± 0.8	81.2 ± 0.7	80.1 ± 0.7	89.5 ± 0.2	88.6 ± 0.3	76.8 ± 1.1	77.0 ± 1.3
	2	81.6 ± 0.7	81.9 ± 0.6	81.1 ± 0.8	78.6 ± 0.6	78.6 ± 0.7	79.5 ± 0.6	89.3 ± 0.3	87.5 ± 0.7	76.2 ± 0.9	76.5 ± 1.4
	3	83.0 ± 0.8	83.8 ± 0.7	80.9 ± 0.8	79.5 ± 0.5	78.6 ± 0.7	79.9 ± 0.7	89.9 ± 0.2	88.8 ± 0.6	75.7 ± 1.1	77.9 ± 0.8
	4	81.8 ± 1.0	84.0 ± 0.5	81.5 ± 0.7	78.9 ± 0.6	78.8 ± 0.5	79.7 ± 0.7	89.1 ± 0.4	87.1 ± 0.4	72.2 ± 0.9	75.3 ± 1.4
8-month	1	80.6 ± 0.9	81.5 ± 0.8	75.6 ± 1.0	76.7 ± 0.9	73.1 ± 0.7	76.0 ± 1.0	86.2 ± 0.5	86.0 ± 0.6	75.4 ± 0.9	75.7 ± 1.2
	2	79.4 ± 0.8	82.2 ± 0.6	75.5 ± 0.8	75.3 ± 1.1	74.7 ± 0.7	76.5 ± 1.2	85.4 ± 0.7	85.8 ± 0.6	74.3 ± 1.4	77.5 ± 1.1
	3	80.9 ± 1.0	82.6 ± 0.8	75.3 ± 0.9	75.4 ± 0.9	75.0 ± 0.7	74.0 ± 1.0	86.0 ± 0.2	84.6 ± 0.8	77.1 ± 0.8	76.6 ± 1.2
	4	80.2 ± 1.0	82.6 ± 0.7	76.8 ± 0.9	75.2 ± 1.1	74.6 ± 0.6	75.1 ± 1.1	85.3 ± 0.5	85.4 ± 0.8	75.5 ± 1.1	76.5 ± 0.9
**Males**											
3-month	1	84.8 ± 0.3	86.9 ± 0.5	81.2 ± 0.6	79.7 ± 0.9	81.2 ± 0.7	80.1 ± 0.7	83.1 ± 1.1	86.7 ± 0.8	73.6 ± 1.2	73.5 ± 1.4
	2	85.1 ± 0.5	86.2 ± 0.5	80.6 ± 0.6	80.2 ± 0.6	80.0 ± 0.7	81.3 ± 0.5	83.4 ± 1.1	86.7 ± 0.7	72.4 ± 1.5	72.9 ± 1.1
	3	85.3 ± 0.5	86.6 ± 0.4	80.8 ± 0.8	79.6 ± 0.6	80.2 ± 0.8	81.6 ± 0.7	86.9 ± 0.7	86.6 ± 0.8	76.3 ± 1.1	71.3 ± 1.5
	4	84.8 ± 0.6	85.8 ± 0.4	79.5 ± 0.5	79.1 ± 0.7	80.4 ± 0.6	80.4 ± 1.0	85.8 ± 1.1	86.8 ± 0.7	72.8 ± 1.6	72.7 ± 1.7
8-month	1	79.7 ± 0.8	82.0 ± 0.7	77.1 ± 0.6	74.5 ± 1.4	74.4 ± 0.9	75.6 ± 1.1	69.2 ± 2.8	82.6 ± 1.1	68.8 ± 1.8	68.3 ± 1.7
	2	78.5 ± 0.6	82.6 ± 0.8	76.5 ± 0.9	76.8 ± 0.8	74.2 ± 1.2	74.9 ± 1.1	82.2 ± 0.9	84.3 ± 0.9	65.0 ± 1.6	61.3 ± 2.3
	3	79.1 ± 0.7	81.4 ± 0.7	77.6 ± 0.7	77.2 ± 1.2	71.9 ± 0.7	73.1 ± 0.9	81.7 ± 1.9	83.1 ± 1.2	72.2 ± 1.9	65.0 ± 2.7
	4	80.3 ± 0.9	80.7 ± 0.8	78.1 ± 0.8	75.1 ± 1.4	71.7 ± 1.2	73.0 ± 1.1	79.6 ± 1.9	82.6 ± 1.0	71.2 ± 1.3	64.0 ± 2.3

All values = mean ± SEM.
N = 16–18 for each strain/sex/cage/density group.
[a] For details of floor space for each density group, see Table 1.
