# Supplementary material for: Effects of Housing Density in Five Inbred Strains of Mice
Source: PLoS One. 2014 Mar 21;9(3):e90012. doi: 10.1371/journal.pone.0090012 (PMC3962340; doi:10.1371/journal.pone.0090012)
Supplement: Table S10 — Neutro,Reticulocyes131029. Neutrophil count (% white blood cells) and reticulocyte count (%) for each of 5 strains for both the 3-month and 8-month timeframes. (PDF) [file pone.0090012.s012.pdf]

Table S10. Neutrophil count and reticulocyte count.

Time-frame	Density group[a]	129S1/SvImJ		A/J		BALB/cByJ		C57BL/6J		DBA/2J	
		Duplex	Shoebox	Duplex	Shoebox	Duplex	Shoebox	Duplex	Shoebox	Duplex	Shoebox
**NEUTROPHIL COUNT (% white blood cells)**											
**Females**											
3-month	1	12.3 ± 0.6	10.6 ± 0.5	15.9 ± 0.6	15.7 ± 0.8	14.1 ± 0.7	15.0 ± 0.7	6.3 ± 0.3	7.1 ± 0.4	17.0 ± 0.7	16.4 ± 1.0
	2	12.4 ± 0.6	12.7 ± 0.6	13.9 ± 0.8	16.0 ± 0.5	16.4 ± 0.6	15.7 ± 0.5	6.6 ± 0.3	8.0 ± 0.6	17.5 ± 0.8	16.3 ± 0.9
	3	11.1 ± 0.8	11.1 ± 0.9	14.4 ± 0.8	16.4 ± 0.6	16.6 ± 0.6	15.3 ± 0.7	5.9 ± 0.2	6.9 ± 0.4	17.8 ± 0.9	16.0 ± 0.6
	4	12.3 ± 0.8	10.9 ± 0.4	13.7 ± 0.7	16.6 ± 0.6	16.4 ± 0.6	15.3 ± 0.6	6.7 ± 0.3	8.1 ± 0.5	19.3 ± 0.6	17.3 ± 0.7
8-month	1	12.9 ± 0.8	13.0 ± 0.6	18.4 ± 0.9	17.3 ± 0.9	20.8 ± 0.6	18.2 ± 1.0	8.6 ± 0.3	9.2 ± 0.4	18.4 ± 0.8	17.0 ± 0.9
	2	13.1 ± 0.9	13.0 ± 0.7	18.6 ± 0.8	18.8 ± 1.0	19.7 ± 0.6	17.9 ± 1.1	9.2 ± 0.6	9.1 ± 0.4	18.3 ± 1.0	15.8 ± 0.8
	3	12.9 ± 0.7	12.6 ± 0.7	18.8 ± 0.8	18.8 ± 0.8	19.9 ± 0.7	19.7 ± 1.0	9.0 ± 0.3	10.2 ± 0.7	16.6 ± 0.7	15.9 ± 0.8
	4	13.5 ± 1.1	12.3 ± 0.7	17.7 ± 0.9	19.2 ± 1.0	19.7 ± 0.5	17.6 ± 0.9	9.2 ± 0.4	9.2 ± 0.6	18.0 ± 0.9	16.3 ± 0.8
Males											
3-month	1	11.4 ± 0.3	9.7 ± 0.4	14.6 ± 0.6	15.5 ± 0.7	14.5 ± 0.6	14.9 ± 0.5	11.8 ± 0.9	9.0 ± 0.7	20.1 ± 1.1	18.5 ± 1.0
	2	10.9 ± 0.3	9.8 ± 0.4	14.7 ± 0.6	14.9 ± 0.5	15.1 ± 0.5	13.7 ± 0.4	11.2 ± 1.0	8.8 ± 0.6	20.5 ± 1.2	19.6 ± 1.1
	3	10.9 ± 0.4	9.3 ± 0.2	14.6 ± 0.7	16.1 ± 0.4	15.1 ± 0.7	13.5 ± 0.6	8.3 ± 0.6	8.9 ± 0.7	18.4 ± 1.1	19.1 ± 0.7
	4	11.2 ± 0.5	10.3 ± 0.4	15.3 ± 0.4	16.1 ± 0.7	15.0 ± 0.5	14.3 ± 0.7	9.3 ± 1.0	8.8 ± 0.7	18.5 ± 0.9	18.8 ± 1.2
8-month	1	15.6 ± 0.5	12.8 ± 0.6	17.2 ± 0.6	17.0 ± 0.8	20.0 ± 0.8	17.5 ± 0.5	22.6 ± 2.9	12.9 ± 1.0	23.4 ± 1.5	23.3 ± 1.5
	2	16.2 ± 0.5	12.4 ± 0.6	17.4 ± 0.8	16.5 ± 0.5	20.0 ± 0.9	19.1 ± 0.9	12.3 ± 0.9	11.0 ± 0.8	27.2 ± 1.4	31.4 ± 2.5
	3	15.8 ± 0.5	13.6 ± 0.6	16.3 ± 0.5	16.7 ± 1.0	22.2 ± 0.7	21.0 ± 0.9	11.1 ± 0.9	11.2 ± 0.8	21.5 ± 1.8	27.1 ± 2.5
	4	15.4 ± 0.7	14.2 ± 0.8	16.0 ± 0.7	16.7 ± 0.7	22.1 ± 1.1	20.8 ± 1.0	14.5 ± 2.0	11.3 ± 0.7	22.0 ± 1.2	28.6 ± 2.4
**RETICULOCYTE COUNT (%)**											
**Females**											
3-month	1	2.2 ± 0.09	1.9 ± 0.07	2.6 ± 0.12	2.5 ± 0.13	2.2 ± 0.12	2.5 ± 0.13	2.6 ± 0.05	2.7 ± 0.11	1.7 ± 0.17	2.4 ± 0.17
	2	2.1 ± 0.10	2.2 ± 0.08	2.6 ± 0.09	2.6 ± 0.07	2.0 ± 0.08	2.3 ± 0.11	2.8 ± 0.05	2.9 ± 0.08	2.1 ± 0.19	2.0 ± 0.16
	3	2.0 ± 0.07	2.1 ± 0.09	2.7 ± 0.12	2.5 ± 0.08	1.8 ± 0.12	2.6 ± 0.14	2.9 ± 0.09	2.9 ± 0.09	2.2 ± 0.23	2.2 ± 0.22
	4	2.0 ± 0.09	2.0 ± 0.06	2.6 ± 0.13	2.6 ± 0.11	1.9 ± 0.10	2.5 ± 0.11	2.8 ± 0.05	3.0 ± 0.08	2.5 ± 0.27	2.2 ± 0.13
8-month	1	2.8 ± 0.12	2.8 ± 0.13	3.0 ± 0.24	3.1 ± 0.14	2.4 ± 0.09	3.0 ± 0.12	2.9 ± 0.08	3.6 ± 0.15	2.0 ± 0.19	2.4 ± 0.21
	2	2.7 ± 0.14	2.8 ± 0.13	3.0 ± 0.19	2.9 ± 0.11	2.3 ± 0.09	3.1 ± 0.15	2.9 ± 0.07	3.6 ± 0.07	2.1 ± 0.26	2.5 ± 0.26
	3	2.6 ± 0.19	2.9 ± 0.20	2.9 ± 0.15	3.5 ± 0.14	2.3 ± 0.07	3.2 ± 0.11	2.9 ± 0.06	3.5 ± 0.09	2.4 ± 0.28	2.7 ± 0.23
	4	2.7 ± 0.15	2.6 ± 0.09	3.1 ± 0.16	3.3 ± 0.20	2.3 ± 0.09	3.0 ± 0.13	2.8 ± 0.08	3.7 ± 0.10	2.2 ± 0.23	2.2 ± 0.15
**Males**											
3-month	1	2.2 ± 0.06	2.1 ± 0.06	2.6 ± 0.08	2.5 ± 0.06	2.0 ± 0.07	2.2 ± 0.09	3.0 ± 0.06	3.1 ± 0.06	2.2 ± 0.12	2.1 ± 0.14
	2	2.2 ± 0.08	2.0 ± 0.04	2.8 ± 0.08	2.6 ± 0.07	1.9 ± 0.05	2.1 ± 0.06	2.9 ± 0.05	3.0 ± 0.06	2.5 ± 0.15	2.4 ± 0.14
	3	2.2 ± 0.07	2.0 ± 0.04	2.5 ± 0.08	2.5 ± 0.09	2.1 ± 0.05	2.3 ± 0.06	3.0 ± 0.06	3.1 ± 0.09	2.5 ± 0.16	2.2 ± 0.10
	4	2.3 ± 0.09	2.0 ± 0.04	2.7 ± 0.09	2.4 ± 0.12	2.1 ± 0.06	2.4 ± 0.05	2.9 ± 0.07	2.9 ± 0.07	2.5 ± 0.17	2.1 ± 0.14
8-month	1	2.6 ± 0.04	2.7 ± 0.09	2.9 ± 0.06	2.8 ± 0.06	2.4 ± 0.04	3.1 ± 0.06	2.7 ± 0.09	3.5 ± 0.06	2.4 ± 0.10	2.6 ± 0.07
	2	2.6 ± 0.06	2.7 ± 0.09	3.1 ± 0.07	3.1 ± 0.09	2.5 ± 0.05	3.2 ± 0.08	2.7 ± 0.08	3.5 ± 0.09	2.1 ± 0.20	2.8 ± 0.13
	3	2.6 ± 0.05	2.6 ± 0.05	3.1 ± 0.07	3.3 ± 0.07	2.3 ± 0.04	2.9 ± 0.07	2.6 ± 0.10	3.4 ± 0.09	2.2 ± 0.16	2.7 ± 0.17
	4	2.6 ± 0.06	2.6 ± 0.05	3.3 ± 0.10	3.1 ± 0.07	2.5 ± 0.04	2.9 ± 0.08	2.7 ± 0.07	3.5 ± 0.10	2.1 ± 0.11	2.8 ± 0.13

All values = mean ± SEM.
N = 16–18 for each strain/sex/cage/density group.
[a] For details of floor space for each density group, see Table 1.
