# Supplementary material for: Effects of Housing Density in Five Inbred Strains of Mice
Source: PLoS One. 2014 Mar 21;9(3):e90012. doi: 10.1371/journal.pone.0090012 (PMC3962340; doi:10.1371/journal.pone.0090012)
Supplement: Table S11 — Eosinophils,Monocytes131029. Eosinophil count and monocyte count (% white blood cells) for each of 5 strains for both the 3-month and 8-month timeframes. (PDF) [file pone.0090012.s013.pdf]

Table S11. Eosinophil count and monocyte count.

Time-frame	Density group[a]	129S1/SvImJ		A/J		BALB/cByJ		C57BL/6J		DBA/2J	
		Duplex	Shoebox	Duplex	Shoebox	Duplex	Shoebox	Duplex	Shoebox	Duplex	Shoebox
**EOSINOPHIL COUNT (% white blood cells)**											
**Females**											
3-month	1	2.9 ± 0.2	2.8 ± 0.2	1.3 ± 0.1	1.3 ± 0.1	1.9 ± 0.2	2.1 ± 0.1	1.7 ± 0.1	1.7 ± 0.1	1.7 ± 0.1	1.9 ± 0.1
	2	3.2 ± 0.3	2.9 ± 0.2	1.5 ± 0.1	1.7 ± 0.1	2.2 ± 0.1	2.2 ± 0.2	1.6 ± 0.1	1.8 ± 0.1	1.8 ± 0.1	2.3 ± 0.2
	3	2.7 ± 0.3	2.2 ± 0.2	1.4 ± 0.1	1.3 ± 0.1	1.9 ± 0.2	2.1 ± 0.2	1.7 ± 0.1	1.6 ± 0.1	2.0 ± 0.2	2.0 ± 0.2
	4	3.1 ± 0.2	2.8 ± 0.2	1.4 ± 0.1	1.4 ± 0.1	2.1 ± 0.2	2.5 ± 0.2	1.6 ± 0.1	2.0 ± 0.1	2.0 ± 0.1	2.1 ± 0.2
8-month	1	3.1 ± 0.4	2.4 ± 0.2	1.8 ± 0.1	2.2 ± 0.1	2.7 ± 0.2	2.7 ± 0.2	1.7 ± 0.1	1.9 ± 0.2	2.2 ± 0.2	2.9 ± 0.2
	2	4.0 ± 0.5	2.6 ± 0.2	2.1 ± 0.2	2.3 ± 0.1	2.4 ± 0.2	2.4 ± 0.2	1.8 ± 0.1	1.8 ± 0.1	2.3 ± 0.2	2.3 ± 0.3
	3	3.4 ± 0.4	2.6 ± 0.2	2.0 ± 0.1	2.1 ± 0.1	2.5 ± 0.2	3.1 ± 0.3	1.7 ± 0.1	2.1 ± 0.1	2.3 ± 0.3	2.5 ± 0.2
	4	3.5 ± 0.3	2.8 ± 0.3	1.8 ± 0.1	2.2 ± 0.1	2.4 ± 0.2	3.3 ± 0.2	1.6 ± 0.1	2.0 ± 0.1	2.1 ± 0.2	2.3 ± 0.2
Males											
3-month	1	1.3 ± 0.1	1.2 ± 0.1	1.1 ± 0.1	1.1 ± 0.0	1.6 ± 0.1	2.3 ± 0.2	1.4 ± 0.1	1.8 ± 0.1	1.8 ± 0.1	2.2 ± 0.3
	2	1.3 ± 0.1	1.4 ± 0.1	1.2 ± 0.1	1.4 ± 0.1	2.2 ± 0.1	2.1 ± 0.1	1.5 ± 0.1	1.8 ± 0.1	1.8 ± 0.1	2.1 ± 0.2
	3	1.3 ± 0.1	1.4 ± 0.1	1.1 ± 0.1	1.3 ± 0.1	2.1 ± 0.2	2.0 ± 0.2	1.5 ± 0.1	1.6 ± 0.1	1.5 ± 0.1	1.8 ± 0.1
	4	1.4 ± 0.1	1.5 ± 0.1	1.3 ± 0.1	1.3 ± 0.1	2.1 ± 0.1	2.1 ± 0.1	1.5 ± 0.1	1.9 ± 0.1	2.0 ± 0.1	2.2 ± 0.2
8-month	1	2.2 ± 0.2	2.3 ± 0.1	1.6 ± 0.1	2.4 ± 0.3	2.4 ± 0.2	2.5 ± 0.1	1.7 ± 0.1	2.0 ± 0.2	2.1 ± 0.2	2.7 ± 0.4
	2	2.2 ± 0.1	2.2 ± 0.1	1.8 ± 0.1	2.6 ± 0.3	2.4 ± 0.2	2.8 ± 0.2	2.0 ± 0.1	2.0 ± 0.1	1.7 ± 0.2	2.5 ± 0.1
	3	2.0 ± 0.1	2.4 ± 0.1	1.7 ± 0.1	2.0 ± 0.2	2.6 ± 0.2	2.7 ± 0.2	2.0 ± 0.2	2.1 ± 0.2	1.9 ± 0.2	2.3 ± 0.3
	4	1.9 ± 0.2	2.3 ± 0.1	1.6 ± 0.1	2.5 ± 0.3	2.9 ± 0.2	3.2 ± 0.2	2.1 ± 0.2	2.4 ± 0.2	1.9 ± 0.2	2.7 ± 0.3
**MONOCYTE COUNT (% white blood cells)**											
**Females**											
3-month	1	1.2 ± 0.1	1.3 ± 0.1	2.0 ± 0.1	2.4 ± 0.2	1.4 ± 0.1	1.7 ± 0.1	0.8 ± 0.0	1.5 ± 0.1	2.3 ± 0.2	3.0 ± 0.2
	2	1.4 ± 0.1	1.4 ± 0.1	2.2 ± 0.1	2.3 ± 0.1	1.4 ± 0.1	1.7 ± 0.1	0.8 ± 0.1	1.6 ± 0.1	2.6 ± 0.2	2.6 ± 0.2
	3	1.2 ± 0.1	1.3 ± 0.1	2.1 ± 0.1	2.2 ± 0.1	1.5 ± 0.1	1.8 ± 0.1	0.8 ± 0.0	1.5 ± 0.1	2.2 ± 0.2	2.5 ± 0.1
	4	1.5 ± 0.1	1.2 ± 0.1	2.0 ± 0.1	2.4 ± 0.1	1.4 ± 0.1	1.5 ± 0.1	0.9 ± 0.1	1.7 ± 0.1	2.7 ± 0.2	2.5 ± 0.2
8-month	1	1.6 ± 0.1	1.5 ± 0.1	2.7 ± 0.2	2.8 ± 0.2	2.1 ± 0.1	2.4 ± 0.1	1.1 ± 0.1	1.7 ± 0.2	2.4 ± 0.1	2.8 ± 0.2
	2	1.6 ± 0.1	1.6 ± 0.1	2.7 ± 0.1	2.7 ± 0.2	2.0 ± 0.1	2.2 ± 0.1	1.4 ± 0.1	1.6 ± 0.1	2.5 ± 0.2	2.8 ± 0.2
	3	1.7 ± 0.1	1.6 ± 0.1	2.8 ± 0.1	2.7 ± 0.2	1.7 ± 0.1	2.3 ± 0.1	1.4 ± 0.1	2.0 ± 0.1	2.5 ± 0.2	3.0 ± 0.2
	4	1.8 ± 0.1	1.5 ± 0.1	2.6 ± 0.2	2.7 ± 0.1	2.1 ± 0.1	2.6 ± 0.2	1.2 ± 0.1	1.8 ± 0.1	2.8 ± 0.2	3.2 ± 0.2
**Males**											
3-month	1	1.4 ± 0.1	1.4 ± 0.1	2.0 ± 0.1	2.5 ± 0.2	1.3 ± 0.1	1.9 ± 0.1	1.2 ± 0.1	1.4 ± 0.1	2.6 ± 0.2	3.0 ± 0.2
	2	1.3 ± 0.1	1.3 ± 0.1	2.2 ± 0.1	2.6 ± 0.2	1.3 ± 0.1	1.8 ± 0.1	1.1 ± 0.1	1.6 ± 0.1	2.6 ± 0.1	3.0 ± 0.2
	3	1.3 ± 0.1	1.4 ± 0.1	2.3 ± 0.2	2.5 ± 0.1	1.2 ± 0.1	1.9 ± 0.2	1.0 ± 0.1	1.5 ± 0.1	2.3 ± 0.2	2.8 ± 0.1
	4	1.4 ± 0.1	1.3 ± 0.1	2.7 ± 0.2	2.6 ± 0.2	1.3 ± 0.1	1.7 ± 0.1	1.1 ± 0.1	1.8 ± 0.1	2.7 ± 0.1	2.8 ± 0.2
8-month	1	2.3 ± 0.2	1.9 ± 0.1	3.0 ± 0.2	3.2 ± 0.1	2.3 ± 0.1	2.5 ± 0.1	1.5 ± 0.1	1.6 ± 0.1	2.9 ± 0.2	3.6 ± 0.2
	2	2.2 ± 0.2	1.8 ± 0.1	3.2 ± 0.2	3.2 ± 0.2	2.2 ± 0.1	2.3 ± 0.1	1.2 ± 0.1	1.5 ± 0.1	3.3 ± 0.2	3.3 ± 0.3
	3	2.1 ± 0.1	1.9 ± 0.1	3.4 ± 0.2	3.0 ± 0.2	2.0 ± 0.1	2.3 ± 0.1	1.1 ± 0.1	1.7 ± 0.1	3.0 ± 0.2	3.7 ± 0.3
	4	2.3 ± 0.2	2.0 ± 0.1	2.9 ± 0.2	3.4 ± 0.2	2.3 ± 0.1	2.3 ± 0.1	1.3 ± 0.1	1.9 ± 0.1	3.5 ± 0.2	3.6 ± 0.2

All values = mean ± SEM.
N = 16–18 for each strain/sex/cage/density group.
[a] For details of floor space for each density group, see Table 1.
